# Characterization of B-BOX gene family and their expression profiles under hormonal, abiotic and metal stresses in *Poaceae* plants

**DOI:** 10.1186/s12864-018-5336-z

**Published:** 2019-01-09

**Authors:** Abdullah Shalmani, Xiu-Qing Jing, Yi Shi, Izhar Muhammad, Meng-Ru Zhou, Xiao-Yong Wei, Qiong-Qiong Chen, Wen-Qiang Li, Wen-Ting Liu, Kun-Ming Chen

**Affiliations:** State Key Laboratory of Crop Stress Biology in Arid Areas, College of Life Sciences, Northwest A&F University, Yangling, 712100 China

**Keywords:** *BBX*, *Poaceae*, synteny, expression analysis

## Abstract

**Background:**

B-box **(**BBX) proteins play important roles in plant growth regulation and development including photomorphogenesis, photoperiodic regulation of flowering, and responses to biotic and abiotic stresses.

**Results:**

In the present study we retrieved total 131 *BBX* members from five *Poaceae* species including 36 from maize, 30 from rice, 24 from sorghum*,* 22 from stiff brome, and 19 from Millet. All the *BBX* genes were grouped into five subfamilies on the basis of their phylogenetic relationships and structural features. The expression profiles of 12 *OsBBX* genes in different tissues were evaluated through qRT-PCR, and we found that most rice *BBX* members showed high expression level in the heading stage compared to seedling and booting stages. The expression of *OsBBX1*, *OsBBX2*, *OsBBX8*, *OsBBX19*, and *OsBBX24* was strongly induced by abiotic stresses such as drought, cold and salt stresses. Furthermore, the expression of *OsBBX2*, *OsBBX7*, *OsBBX17*, *OsBBX19*, and *OsBBX24* genes was up-regulated under GA, SA and MeJA hormones at different time points. Similarly, the transcripts level of *OsBBX1*, *OsBBX7*, *OsBBX8*, *OsBBX17*, and *OsBBX19* genes were significantly affected by heavy metals such as Fe, Ni, Cr and Cd.

**Conclusion:**

Change in the expression pattern of *BBX* members in response to abiotic, hormone and heavy metal stresses signifies their potential roles in plant growth and development and in response to multivariate stresses. The findings suggest that *BBX* genes could be used as potential genetic markers for the plants, particularly in functional analysis and determining their roles under multivariate stresses.

**Electronic supplementary material:**

The online version of this article (10.1186/s12864-018-5336-z) contains supplementary material, which is available to authorized users.

## Background

Zinc finger transcription factors (TFs) are one of the most important families in plants. They regulate different plant growth and development processes. Zinc finger TFs are classified into several subfamilies based on the structural and functional features of their individual members. Among them, B-box proteins (BBXs) drew more attention in recent years due to their multiple functions. The BBXs contain one or two conserved B-box domains near to N-terminus and some have an additional CCT (CONSTANS, CO-like, and TIMING Of CAB1) conserved domain near to C-terminal. The B-box domains are divided into two classes, known as B-box1 (B1) and B-box2 (B2). Two B-box conserved domains are recognized on their consensus sequence and the distance between the zinc-binding residues [1]. The segmental duplication and deletion events during evolution result in the differences of the consensus sequences in the two B-box domains [2, 3]. The highly conserved CCT domain is comprised of 42-43 amino acids and is important for the regulation of functional transcription and nuclear protein transport [4].

Recent genome-wide expression studies suggested that the BBX proteins are involved in plant hormone signaling responses. Abscisic acid (ABA) is a phytohormone which is activated when the plants are exposed to different stresses [5]. Microarray analysis detected that the expression pattern of *BBX* genes is different in response to ABA [6, 7]. The microarray study also found that the expression of *BBX32* was up-regulated by the cyclopentenone precursor of JA, 12-oxo-pentadienoic acid (OPDA), but not by JA or MeJA in *Arabidopsis* plants [8]. In addition, it was found that BZS1/*BBX20* integrates signals from brassinosteroids (BR) and light pathways [9]. BRASSINAZOLE RESISTANT 1 (BZR1) is a transcription factor that triggered hypocotyl growth by directly binding to BBX20 [10]. Interestingly, GATA2, a GATA-binding zinc-finger protein stopped hypocotyl growth by reducing BR signaling action [11]. So, it can be postulated that *BBX20* works together with GATA2 in facilitating light and BR crosstalk. Recently, it was reported that *BBX18* play a potential role in the gibberellin (GA) signaling pathway [12]. Molecular and phenotypic studies proved that *BBX18* enhances the hypocotyl growth by up-regulation of bioactive GA levels. Certainly, *BBX18* promotes the activities of GA3ox1 and GA20ox1 metabolic genes but decreased the activities of GA2ox1 and GA2ox8 catabolic genes under light [12]. The involvement of *BBX* genes in the COP/HY5 signaling pathway indicates that *BBX18* may work as an integrator of both GA and COP1/HY5 pathways [13]. In addition, the microarray database showed that the transcript level of 11 *BBX* genes was distinct in rice when the plants were exposed to auxin, GA, and cytokinin treatments, and the studied rice *BBXs* have hormone-responsive cis-acting elements in their promoters [14]. These results indicate the probable involvement of BBX proteins in hormones signaling in plants. However, the functional mechanisms of BBXs in hormonal signaling pathways are still little known.

BBXs might also play vital roles in abiotic stress tolerance of plants. The salt tolerance protein (STO, *AtBBX24*) enhances the growth of root under a high salinity condition in *Arabidopsis* [15], and was also triggered by the salt tolerance activities in yeast cells [16]. STO inoculates with CLONE EIGHTY-ONE/RADICAL-INDUCED CELL DEATH1 (CEO/RCD1) [17], which negatively regulates a wide range of stress-related genes [18]. Another *BBX* gene, *AtBBX18*, acts as a negative regulator both in photomorphogenesis and thermotolerance in *Arabidopsis* [12]. Furthermore, *AtBBX18* negatively regulates the expression of heat-responsive genes such as *DGD1*, *Hsp70*, *Hsp101*, and *APX2*, thereby reducing germination and seedling survival after the heat treatment [12]. In *Chrysanthemum, CmBBX24* performed a dual function, delaying flowering and also increase cold or drought tolerance [19]. Moreover, the overexpression of *AtBBX24* enhances salt tolerance compared to wild-type plants, and a significant increase in root length in *Arabidopsis* [15]. Twenty-nine out of 30 rice *BBX* genes possess at least one stress-responsive cis-elements such as ARE, Wbox, GC-motif, Box-W1, HSE, and MBS, signifying that these genes may express during biotic and abiotic stresses [14].

The studies on B-box proteins have emerging role in the plant development and of great interest for various researchers nowadays. Although, the *BBX* gene family and their expression patterns under a few hormones were previously reported in rice [14], the evolutionary relationships of BBXs especially in *Poaceae* not yet been clearly understood. Additionally, the roles of *BBX* genes in evolutionary origin and structural changes, the plant stress response and functional diversity of these proteins are still little understood in land plants. Therefore, in the present study, the *BBX* gene family members in five *Poaceae* species and their expression profiles under various hormones, abiotic and heavy metal stresses in rice were systematically investigated. The obtained results will enlighten the novel insights into their action and the evolutionary significance of their functional divergence. Furthermore, the gene expression pattern will assist to improve the potential *BBX* candidate genes involved in plant development regulation and multivariate stress responses.

## Materials

### Identification of *BBX* gene family member

The *Arabidopsis BBX* gene family has already been reported [1]. All the downloaded BBX protein sequences from *Arabidopsis* Information Resource (TAIR) database (http://www.arabidopsis.org) were used as queries for BLASTP search with default parameters against maize genome database (https://maizegdb.org), the rice genome database (Rice Annotation Project (RAP) v1.0, http://rapdb.dna.affrc.go.jp/) and plant genome database (http://plantgdb.org/SbGDB/SiGDB/BdGDB/). Afterward, all the protein sequences were further scanned to check their completeness and presence of the target domain via the following online tools: SMART (http://smart.embl-heidelberg.de/) [20], Inter Pro Scan program (http://www.ebi.ac.uk/interpro/), Conserved Domain Database (CDD) (http://www.ncbi.nlm.nih.gov/cdd/), and Scan Prosite (http://prosite.expasy.org/scanprosite/). The chemical features of BBX proteins such as isoelectric point (PI), molecular weight (kD), instability index, aliphatic index, grand average of hydropathy (GRAVY) and major amino acids of each BBX proteins were investigated using the ExPASy proteomics server (http://web.expasy.org/protparam/)

### Chromosomal localization, Exon and Intron Distribution and Conserved Motif Analysis

The corresponding genome database was used to obtain the candidate *BBX* gene annotations and their chromosomal locations. The exact locations of genes on chromosomes were identified by using MapDraw. The conserved and shared domains for all BBX protein sequences were created by online version 4.9.1 of the Multiple Expectation for Motif Elicitation (MEME) tool (http://meme-suite.org/) [21, 22]. Gene Structure Display Server (http://gsds.cbi.pku.edu.cn) was used to construct the exon-intron structure consisting exon positions and gene length of *BBX* genes.

### Sequence alignment and Phylogenetic analysis

Multiple alignments of BBX protein sequences were performed with DNAMAN software (Version 5.2.2, LynnonBiosoft, Canada), and the sequence logos were constructed through online Weblogo platform (http://weblogo.berkeley.edu/logo.cgi). The candidate BBX proteins were initially multiply aligned by using the ClustalW v2.0 online tool (http://www.ebi.ac.uk/Tools/webservices/services/msa/clustalw2_soap) to further search the evolutionary relationships of the *BBX* gene family and then the maximum likelihood phylogenetic tree was constructed by using the MEGA 6.06 software package with default parameters and the reliability of interior branches was assessed with 1000 bootstrap repetitions.

### Tandem Duplication and Synteny Analysis

The Plant Genome Duplication Database (http://chibba.agtec.uga.edu/duplication/) was used to obtain syntenic blocks. Then circos version software (http://circos.ca/) was used to draw the diagrams. The physical location of a gene on the chromosome was used to find out the Tandem duplication of *BBX* gene. Genes having an adjacent homologous *BBX* gene on the same chromosome with no more than one intervening gene were considered to be tandemly duplicated.

### Plant Material and Growth Conditions

The experimental work was performed in the field of State Key Laboratory of Crops Stress Biology for Arid Areas (Northwest A&F University, Yangling, China). First of all, the seeds were sterilized with 0.5% (w/v) sodium hypochlorite (NaClO) for 4 h, then washed thrice with distilled water. Seeds were then soaked in water for 48 h in darkness. Subsequently, the seeds were propagated on humid cheesecloth at 28 ^°^C for 72 h and wetted with deionized water each day. Healthy and uniform seedlings were selected and grown in hydroponic solution prepared in Milli-Q water [23], containing 16 mM KNO_3_, 6 mM Ca(NO_3_)_2_·4H_2_O, 4 mM NH_4_H_2_PO_4_, 2 mM MgSO_4_·7H_2_O, 50μM KCl, 25μM H_3_BO_3_, 25μM Fe-EDTA, 2μM MnSO_4_·4H_2_O, 2μM ZnSO_4_, 0.5μM Na_2_MoO_4_·2H_2_O, and 0.5μM CuSO_4_·5H_2_O. The plants were floated in nutrient solution fixed with foam plugged in vessels (one plant in the single vessel). The nutrient solutions were continuously aerated and the environment was firmly controlled in growth chamber condition at (16 h/8 h day/night, temperature cycle of 30^°^C /25^°^C, 800 μmol m^–2^ s^–1^ light intensity and 60–65% relative humidity level). The solution was changed after 24 h duration and the pH was adjusted to 5.8 by using NaOH or HCl.

### Stress Treatments and Sample Collection

To identify the transcript profiles of *BBX* genes in rice, the young seedling (two-week-old) were exposed to various abiotic stresses, phytohormones and heavy metals. For heat stress treatment, the seedlings at four-leaf stage were subjected at 40^°^C temperature with 60% humidity, 16 h photoperiod in the growth chamber under fluorescent light for 24 h. For cold stress, at the same stage seedlings were transferred into the cold cabinet (SANYO) under a 14-h light: 10-h dark, with light conditions of 300 μmol photons m^−2^ s^−1^. For dehydration 20% polyethylene glycol (PEG-6000), the solution was purified by passing it through an ion exchange column to remove any impurities and was filtered using Miracloth (22–25 μm, Thomas Scientific, Swedesboro, NJ, USA). Salt (200 mM NaCl) was prepared from stock solution by dissolving in water. Then seedlings were submerged in 20% PEG6000 or 200 mM NaCl solutions for drought and salt treatments respectively. The final hormonal concentration of gibberellic acid (GA) (100 μM), abscisic acid (ABA) (100 μM), methyl jasmonate (MeJA) (100 μM) and salicylic acid (SA) (500 μM) were prepared from stock solutions, after addition of wetting agent Tween-20 at 0.05% (v/v) the individual hormone were sprayed on two weeks old rice leaves. For metals treatments, FeSO_4_ (7 mM), CdCl_2_ (0.5 mM), K_2_Cr_2_O_7_ (1 mM), and NiCl_2_ (1 mM) were prepared from stock solutions and applied into fresh nutrient solution and as [24] with exception of phosphorus (P) that prevents precipitation of lead (Pb) [25]. The whole leaf blades from the treated two-week-old rice plants were harvested at 0h, 3h, 6h, 12h and 24h time intervals after treatments. Rice plants were allowed to grow in normal condition (day/night temperature cycle of 32^°^C /26^°^C, 16 h/8 h photo-period with 800 μmol m ^–2^ s ^–1^ light intensity and 60% humidity), and the different plant organs at various developmental stages (namely seedling, tillering, booting and heading stages) were collected for the analysis of tissue-specific expression. The samples were immediately frozen in liquid nitrogen and stored at -80^°^C until for further analysis.

### Quantitative PCR analysis

The total RNA was extracted from all the samples by using the cetyltrimethylammonium bromide (CTAB) method [26]. The samples were run on 2% agarose gels to examine the intensity of ribosomal RNA (rRNA) bands, degraded products, and gDNA contamination. The residual genomic DNA was removed by treating the RNA samples with RNase-free DNase. The cDNA was synthesized through the PrimeScript RT Reagent Kit with gDNA Eraser (Takara Bio, Shiga, Japan) following the manufacturer’s instructions. All the primers were designed from rice *BBX* sequences for real-time PCR using primer 6.0 (Additional file 1: Table S3). Each primer pair was examined through standard RT-PCR to confirm the size of the amplified product through 1% agarose gel electrophoresis. Real-time PCR was carried out in an iCycler iQ Real-Time PCR Detection System (Bio-Rad). Each reaction consisted of 5 μl SYBR Premix ExTaq (Takara, Kyoto, Japan), 2 μl cDNA samples, and 0.5 μl of each primer (10 μM) and 2 μl ddH_2_O in a reaction system of 10 μl. The thermal cycle was as follows: 95^°^C for 3 min, followed by 40 cycles at 94^°^C for 15 s, 62^°^C for 20 s, and 72^°^C for 20 s. Melting-curve analysis was performed directly after real-time PCR to verify the presence of gene-specific PCR products. This analysis was done by 94^°^C for 15 s, followed by a constant increase from 60 to 95^°^C at a 2% ramp rate. The rice actin gene (*OsActin1*, Gene ID: KC140126) was used as an internal control and served as a standard gene for normalizing all mRNA expression levels. The relative amount of template present in each PCR amplification mixture was evaluated by using the 2−ΔΔCt method.

### Statistical analysis

The data underwent an analysis of variance. The means and standard deviation of three replications for all the treatments were compared by the least significant difference (LSD) test at the 5% level using the SPSS 11.5 software package (SPSS, Chicago, IL, USA). Graphs were designed using Origin 7.5 (Microcal Software Inc., Northampton, MA, USA).

## Results

### Identification, Classification, and Annotation of *BBX* Family Members

The *Arabidopsis BBX* genes were used as quarries sequences against the Hidden Markov Model (HMM) algorithm [27] to retrieve and characterize the *BBX* gene family members in five *Poaceae* species. A total of 131 *BBX* genes were identified in the studied five *Poaceae* species. The number of *BBX* genes members were diverse among these plants such as 36, 30, 24, 22 and 19 *BBX* genes from maize *(Zea mays),* rice *(Oryza sativa),* Sorghum *(Sorghum bicolor),* stiff brome (*Brachypodium distachyon*) and Millet (*Setaria italica*), respectively (Table 1). The potential domains of *BBX* gene family were confirmed through the conserved domain database, Pfam and SMART databases and structural integrity of these domains were drawn by Web Logo and EXPASY-PROSITE. All the putative *BBX* members lack transmembrane segment except *ZmBBX30* (Additional file 1: Figure S1). Moreover, the physiochemical characteristics and amino acid sequence of *BBX* members were studied through EXPASY PROTOPARAM (http://www.expasy.org/tools/protparam.html) online tool (Additional file 1: Table S1). The assumed length of the BBX proteins and molecular weights vary widely, ranging from 9.51 (*OsBBX20*) to 52.89 kD (*SbBBX10*). The maximum number of *Poaceae* BBX proteins was acidic in nature according to their isoelectric point, which was lower than seven. However, the isoelectric point of some *BBX* members (*OsBBX15*, *OsBBX20*, *OsBBX21*, *ZmBBX17*, *ZmBBX19*, *ZmBBX24*, *BdBBX16*, *SbBBX6,* and *SbBBX11*) was greater than seven, indicating that they are alkaline proteins in nature. The present study divided the majority of *Poaceae BBX* genes into unstable proteins because the instability index of most of the genes of this family was greater than 40. However, the instability index of *BdBBX20*, *OsBBX12,* and *OsBBX20* were less than 40, and they corresponded to stable proteins. All the BBX proteins were found to be hydrophilic except *OsBBX25* based on their GRAV value. *ZmBBX9* showed high negative and positive charge residues. Based on a total number of atoms, *SbBBX10* contained the highest number of atoms (7281), followed by *ZmBBX3* (7200). *OsBBX20* was the smallest protein (1299) on the basis of atom compositions. This investigation found that 68 BBX proteins were located on the sense strand, and the remaining 63 BBX proteins were found on the antisense strand. The GC content of the majority studied *BBX* was above 60%. Furthermore, the aliphatic index values ranged from lowest (39.91) (*BdBBX11*) to 78.93 (*SbBBX11*). The major amino acid of the BBX proteins is Ala, followed by Ser, while other most abundant amino acids are Pro, Asp, Asn, or Thr, varied depending on the particular BBX protein (Additional file 1: Table S1).

**Table 1:** Nomenclature, identification, chromosomal location, CDS, and peptide length and weight of *BBX* gene family in *Poaceae* species

Name	Id	Location	Genomic	CDS	Protein	Strand	GC%
ZmBBX1	Zm00001d029149	Chr1: 60531179-60535397	4219	1191	397	+	63.0
ZmBBX2	Zm00001d031662	Chr1: 197918030-197921539	3510	1467	489	-	67.7
ZmBBX3	Zm00001d033719	Chr1: 272190270-272192279	2010	1413	471	+	67.2
ZmBBX4	Zm00001d002806	Chr2: 23197620-23201256	3636	774	258	-	68.2
ZmBBX5	Zm00001d003162	Chr2: 34080161-34081769	1608	960	320	-	72.0
ZmBBX6	Zm00001d006198	Chr2: 201392406-201400007	7601	762	254	+	48.9
ZmBBX7	Zm00001d007107	Chr2: 222393488-222401395	7907	1227	409	-	70.8
ZmBBX8	Zm00001d039437	Chr3: 4316527-4318099	1572	1077	359	+	74.3
ZmBBX9	Zm00001d049347	Chr4: 27315744-27316583	839	837	279	-	72.3
ZmBBX10	Zm00001d051018	Chr4: 137141478-137142700	1222	759	253	-	69.0
ZmBBX11	Zm00001d051047	Chr4: 138655083-138656121	1038	942	314	+	75.3
ZmBBX12	Zm00001d051309	Chr4: 152948011-152949738	1727	831	277	+	71.7
ZmBBX13	Zm00001d051610	Chr4: 164536434-164541204	4770	1410	470	-	46.7
ZmBBX14	Zm00001d051684	Chr4: 166653210-166659390	6180	1218	406	+	46.7
ZmBBX15	Zm00001d013443	Chr5: 11710780-11712488	1708	1239	413	-	68.1
ZmBBX16	Zm00001d014765	Chr5: 62282614-62284983	2369	1065	355	-	69.5
ZmBBX17	Zm00001d015434	Chr5: 89924027-89924707	680	678	226	-	79.8
ZmBBX18	Zm00001d017176	Chr5: 188032923-188034708	1785	1005	335	+	74.5
ZmBBX19	Zm00001d017412	Chr5: 195189894-195191367	1473	426	142	+	72.4
ZmBBX20	Zm00001d017885	Chr5: 209614260-209620254	5994	1383	461	+	48.1
ZmBBX21	Zm00001d017939	Chr5: 210407150-210409118	1968	1395	465	-	67.5
ZmBBX22	Zm00001d036214	Chr6: 77322780-77327461	4681	1158	386	+	50.8
ZmBBX23	Zm00001d036418	Chr6: 87645024-87646346	1322	807	269	+	72.0
ZmBBX24	Zm00001d036676	Chr6: 97378208-97378813	605	603	201	-	81.1
ZmBBX25	Zm00001d037327	Chr6: 121369894-121371711	1817	1356	452	+	69.5
ZmBBX26	Zm00001d037735	Chr6: 135623653-135625596	1943	966	322	-	72.6
ZmBBX27	Zm00001d021278	Chr7: 147632776-147636889	4113	618	206	+	50.7
ZmBBX28	Zm00001d045323	Chr9: 18935792-18946869	1077	1266	422	-	51.8
ZmBBX29	Zm00001d045661	Chr9: 32056769-32058471	1702	1368	456	+	68.1
ZmBBX30	Zm00001d045735	Chr9: 36009335-36013889	4554	1284	428	+	61.1
ZmBBX31	Zm00001d045804	Chr9: 40102185-40108023	5838	1308	436	-	43.9
ZmBBX32	Zm00001d046925	Chr9: 111032442-111034142	1700	1092	364	+	70.5
ZmBBX33	Zm00001d024200	Chr10: 55274175-55275011	836	834	278	-	72.4
ZmBBX34	Zm00001d024213	Chr10: 56996605-56997874	1269	780	260	-	71.7
ZmBBX35	Zm00001d025770	Chr10: 129045322-129046879	1557	969	323	+	72.6
ZmBBX36	Zm00001d025957	Ch10: 134741085-134742626	1541	768	256	+	68.0
OsBBX1	Os01g0202500	chr01:5639835..5641475	1440	1065	355	+	72.4
OsBBX2	Os02g0176000	chr02:4150302..4150970	669	669	223	-	77.4
OsBBX3	Os02g0178100	chr02:4315391..4316956	1370	996	332	+	73.5
OsBBX4	Os02g0606200	chr02:23759252..23760433	1078	816	272	+	71.3
OsBBX5	Os02g0610500	chr02:23989803..23991271	1388	999	333	+	73.7
OsBBX6	Os02g0646200	chr02:26027785..26029488	1198	810	270	+	71.3
OsBBX7	Os02g0724000	chr02:30094300..30099072	2023	1224	408	+	46.2
OsBBX8	Os02g0731700	chr02:30473739..30475800	1469	1044	348	-	64.4
OsBBX9	Os03g0351100	chr03:13153018..13155544	1638	1212	404	+	61.5
OsBBX10	Os03g0711100	chr03:28686958..28689501	1840	1266	422	+	73.6
OsBBX11	Os04g0493000	chr04:24648004..24648863	765	555	185	-	66.3
OsBBX12	Os04g0497700	chr04:24889983..24891483	1394	1002	334	+	74.9
OsBBX13	Os04g0540200	chr04:27027267..27029421	1220	753	251	+	70.3
OsBBX14	Os05g0204600	chr05:6514746..6517280	2058	1137	379	+	72.2
OsBBX15	Os06g0103000	chr06:209204..210107	904	672	223	+	56.8
OsBBX16	Os06g0152200	chr06:2695460..2699468	1449	1083	361	+	52.1
OsBBX17	Os06g0264200	chr06:8704897..8706342	1446	1446	482	+	71.7
OsBBX18	Os06g0275000	chr06:9336376..9338569	1557	1188	396	+	58.0
OsBBX19	Os06g0298200	chr06:11070174..11076691	2201	1227	409	-	47.3
OsBBX20	Os06g0654900	chr06:26843118..26843680	563	246	82	-	69.1
OsBBX21	Os06g0661200	chr06:27253336..27254108	773	726	242	+	78.0
OsBBX22	Os06g0713000	chr06:30196103..30197569	1380	927	309	+	74.0
OsBBX23	Os07g0667300	chr07:28184879..28187843	1547	1143	381	-	64.4
OsBBX24	Os08g0178800	chr08:4610545..4612918	1402	846	282	-	73.0
OsBBX25	Os08g0249000	chr08:9098485..9099878	1025	543	181	+	76.2
OsBBX26	Os08g0536300	chr08:26792942..26797114	1891	1467	489	-	63.8
OsBBX27	Os09g0240200	chr09:3048085..3064471	1362	1008	336	-	66.7
OsBBX28	Os09g0509700	chr09:19783524..19786772	1813	1296	432	-	64.3
OsBBX29	Os09g0527900	chr09:20646416..20649984	1248	636	212	-	49.0
OsBBX30	Os12g0209200	chr12:5699790..5702413	792	633	211	+	71.5
SbBBX1	Sb01g010420	Chr1: 9244438-9245891	2454	1260	420	-	59.0
SbBBX2	Sb01g035400	Chr1: 58949641-58952463	3823	1239	413	-	49.7
SbBBX3	Sb02g030690	Chr2: 65720371-65723158	3788	633	211	+	44.8
SbBBX4	Sb02g042230	Chr2: 75932483-75934993	3511	1239	413	-	53.0
SbBBX5	Sb03g002510	Chr3: 2287972-2290172	3201	1056	352	-	56.5
SbBBX6	Sb04g003470	Chr4: 3321373-3324297	3925	1458	486	+	49.6
SbBBX7	Sb04g005250	Chr4: 5107155-5108908	2754	1128	376	+	50.1
SbBBX6	Sb04g025400	Chr4: 55156181-55157415	2235	789	263	-	56.8
SbBBX7	Sb04g025660	Chr4: 55434304-55435890	2587	1008	336	+	56.3
SbBBX8	Sb04g028920	Chr4: 58992284-58994122	2839	1464	488	+	58.8
SbBBX9	Sb04g029180	Chr4: 59189963-59191466	2504	867	289	+	40.6
SbBBX12	Sb04g029480	Chr4: 59566100-59571089	5990	1218	406	-	45.9
SbBBX10	Sb04g033440	Chr4:63334637-63336646	3010	885	295	-	52.2
SbBBX11	Sb06g021170	Chr6: 50527547-50528989	2443	804	268	-	55.1
SbBBX12	Sb06g021480	Chr6:50736218-50737513	2296	987	329	+	60.2
SbBBX13	Sb06g023960	Chr6: 53024486-53026365	2880	792	264	+	52.0
SbBBX14	Sb07g004973	Chr7: 6615154-6617300	2869	813	271	-	52.2
SbBBX15	Sb07g025940	Chr7: 61088100-61092224	5125	1470	490	+	51.2
SbBBX16	Sb08g006510	Chr8: 10161992-10163814	2832	735	245	+	46.7
SbBBX17	Sb09g006370	Chr9: 9921800-9923032	2233	1008	336	+	63.3
SbBBX18	Sb10g003680	3189527-3194818	6292	1125	375	+	44.4
SbBBX19	Sb10g009480	Chr10: 10694648-10696491	2844	1422	474	+	57.0
SbBBX20	Sb10g010050	Chr10: 12275128-12276617	2490	1233	411	+	50.1
SbBBX21	Sb10g010860	Chr10: 14421774-14424602	3829	1218	406	-	39.8
SbBBX22	Sb10g026060	Chr10: 59584561-595898674	2801	1128	376	-	58.9
SbBBX23	Sb10g029840	Chr10: 55386280-55388080	2484	915	305	-	53.8
SbBBX24	Sb10g002725	Chr10: 59584561-595898674	2354	900	300	-	50.4
BdBBX1	Bradi1g11310	Chr1: 8356244-8358332	2089	1269	423	+	60.0
BdBBX2	Bradi1g31280	Chr1: 26748157-26749884	2928	1044	348	+	55.2
BdBBX3	Bradi1g35030	Chr1: 30557402-30558987	2789	867	289	-	53.4
BdBBX4	Bradi1g43220	Chr1: 40823689-40831526	5023	1134	378	+	40.9
BdBBX5	Bradi1g43670	Chr1: 41483191-41486564	4578	1308	436	-	40.2
BdBBX6	Bradi1g43990	Chr1: 41909639-41911589	3151	1101	367	-	57.3
BdBBX7	Bradi1g49260	Chr1: 48014263-48018866	5804	1134	378	-	44.4
BdBBX8	Bradi1g62420	Chr1: 61651141-61654384	4444	1056	352	-	54.1
BdBBX9	Bradi2g06370	Chr2: 4839844-4841420	2777	999	333	+	57.1
BdBBX10	Bradi2g32900	Chr2: 32830551-32831957	2607	1137	379	-	53.8
BdBBX11	Bradi3g05800	Chr3: 4124615-4126380	2966	693	231	+	55.9
BdBBX12	Bradi3g15490	Chr3: 13785466-13787484	3219	1470	490	+	48.8
BdBBX13	Bradi3g41500	Chr3: 43415799-43419719	5121	774	258	-	49.9
BdBBX14	Bradi3g48180	Chr3: 49677419-49678624	2405	1239	413	-	54.2
BdBBX15	Bradi3g56260	Chr3: 56219290-56221877	3788	1179	393	+	41.1
BdBBX16	Bradi3g56490	Chr3: 56348167-56352072	5106	1344	448	-	41.7
BdBBX17	Bradi3g57000	Chr3: 56695125-56696699	2345	633	211	+	49.4
BdBBX18	Bradi4g35950	Chr4: 41215132-41218558	4627	666	222	+	44.0
BdBBX19	Bradi4g40250	Chr4: 44678780-44680513	2934	801	267	-	45.1
BdBBX20	Bradi5g14280	Chr5: 17712355-17713552	3908	1023	341	+	48.4
BdBBX21	Bradi5g14600	Chr5: 18046925-18048712	2988	801	267	+	60.8
BdBBX22	Bradi5g17080	Chr5: 20311769-20313350	2782	801	267	+	53.5
SiBBX1	Si017487m	Chr1: 6104121-6106008	3088	1158	386	-	74.8
SiBBX2	Si018081m	Chr1: 32838588-32840495	3108	807	269	+	71.7
SiBBX3	Si017374m	Chr1: 36700617-36705652	6236	1221	407	+	45.8
SiBBX4	Si019803m	Chr1: 36911502-36913121	2820	1065	355	-	45.6
SiBBX5	Si017124m	Chr1: 37159623-37161711	3289	1395	465	-	70.8
SiBBX6	Si030034m	Chr2: 26392218-26396291	5274	1218	406	+	47.5
SiBBX7	Si031264m	Chr2: 38047130-38051532	5603	531	177	+	45.3
SiBBX8	Si022650m	Chr3: 4644038-4645844	3007	978	326	+	73.9
SiBBX9	Si024510m	Chr3: 6380462-6382407	3146	654	218	-	71.3
SiBBX10	Si006670m	Chr4: 5544882-5549372	5691	1134	378	-	53.3
SiBBX11	Si006432m	Chr4: 11409189-11411174	3186	1335	445	+	72.1
SiBBX12	Si006690m	Chr4: 31034615-31036532	3118	1116	372	+	70.0
SiBBX13	Si006993m	Chr4: 39392572-39394067	2696	903	301	-	71.9
SiBBX14	Si001636m	Chr5: 12649122-12651152	3231	1239	413	+	73.0
SiBBX16	Si014382m	Chr6: 33985026-33988379	3354	705	235	-	49.0
SiBBX17	Si014037m	Chr6: 33985026-33989297	5472	1008	336	-	49.0
SiBBX15	Si010884m	Chr7: 23710294-23711500	2407	774	258	-	70.2
SiBBX16	Si010592m	Chr7: 23922376-23923928	2753	978	326	+	74.2
SiBBX17	Si010885m	Chr7: 26130036-26131926	3091	774	258	+	69.7
SiBBX18	Si035937m	Chr9: 7441605-7443503	3099	1218	406	-	70.6
SiBBX19	Si034611m	Chr9: 46372657-46376370	4914	1944	648	-	64.8

### Systematic Evolutionary Relationship, Gene Structural Diversity, and Motif Analysis

**Fig. 1:**
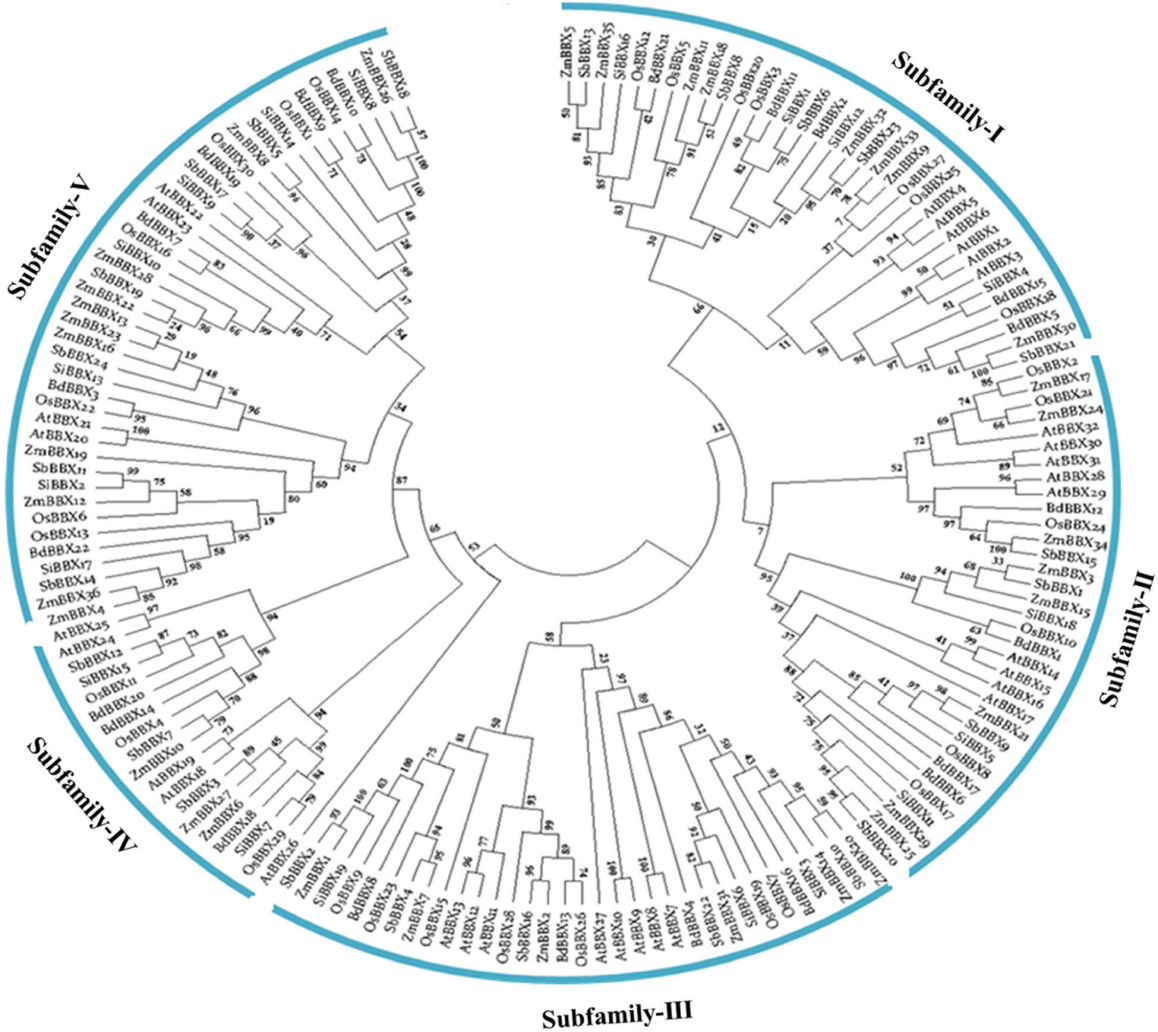
Systematic evolutionary relationships of *BBX* gene family five different *Poaceae* species and *Arabidopsis* among five lineages within the subgroup. The five conserved subfamilies are marked by different numbers and represented as subfamily-I, II, III, IV and V

We found four different classes of BBX proteins on the basis of domain organization; BBXs containing one B-box domain, BBXs having two B-boxes domains, BBXs possessing one B-box and additional CCT domain, and BBXs with two B-boxes and additional CCT domain (Table 2). The homologs of *BBX* genes from six different species were selected for the multiple sequence alignments and phylogenetic relationships analysis to study the evolutionary phylogenetic relationships and functional divergence among *BBX* genes (Fig. 1). We constructed an unrooted maximum-likelihood phylogenetic tree using MEGA 6.06 Software to investigate the evolutionary relationship. The present study clustered the *BBX* genes into five well-conserved subfamilies based on the difference of protein topological structure with high bootstrap support (Fig. 1). The phylogenetic tree divided the *BBX* from five *Poaceae* plants (maize*,* rice*,* sorghum*,* stiff brome, and millet) and one model plant (Arabidopsis) into five subfamilies based on our analysis. Maximum numbers of *BBX* genes containing only one B-box domain were found in subfamily II, IV and V. Most of *BBX* genes with two B-boxes domains were clustered into subfamily V and IV. The third class of *BBX* genes, containing one B-box and additional CCT domain were observed in subfamily I, II and III. Two B-box possessing genes with additional CCT domain were grouped to subfamily I and III. Furthermore, we also evaluated the *Arabidopsis BBX* genes to study their phylogenetic relationship with *Poaceae BBX* members. We found quite a similar clustering for *Arabidopsis BBX* genes with *Poaceae BBX* genes in this study (Fig. 1). *Arabidopsis BBX* possessing only one B-box domain was detected in subfamily II and IV. Two B-boxes domains containing *AtBBXs* were grouped into IV and V. *AtBBXs* with one B-box and additional CCT domain containing genes were detected in subfamily II, whereas two B-boxes and additional CCT possessing *AtBBXs* genes were noted in subfamily I and III.

**Table 2:**
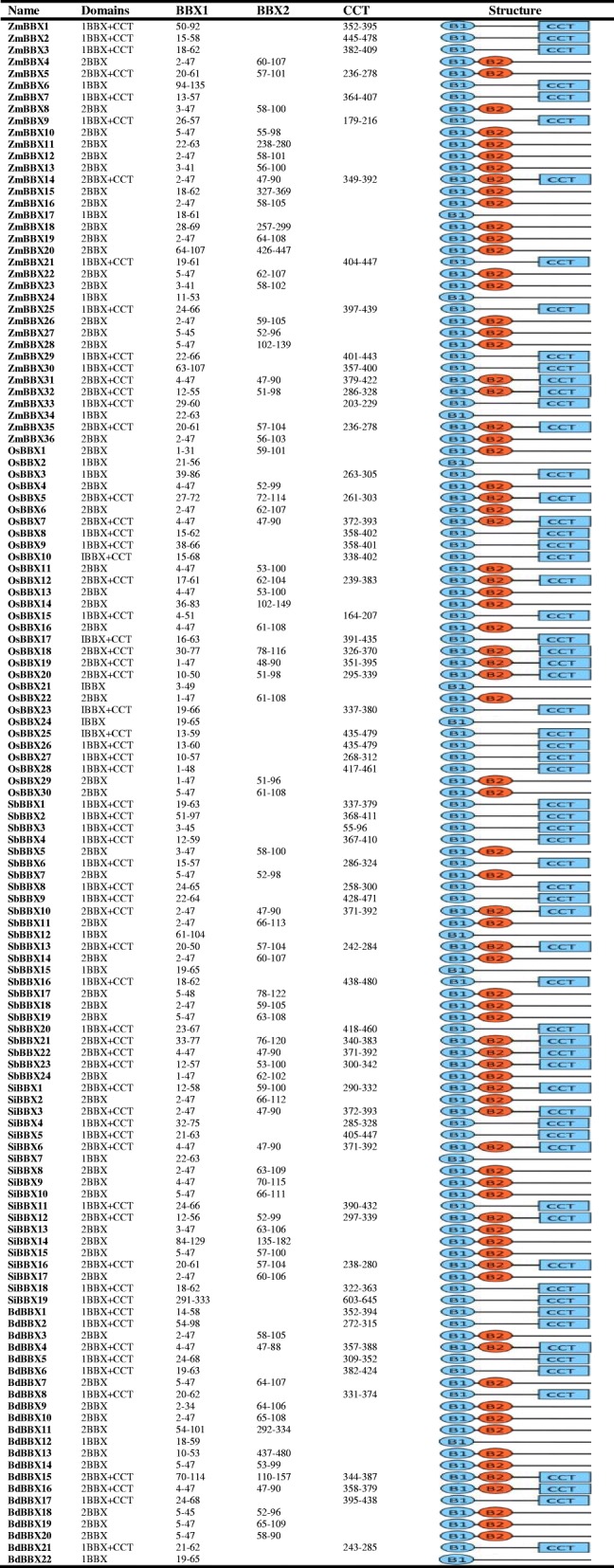
Structures of the BBX proteins. The length and order of the domains represent their actual location within each protein

*Abbreviations*: *B1* B-box1, *B2* B-box2

The conservation of gene structure in a paralogous gene is sufficient to determine the evolutionary connection between introns in various circumstances; therefore, an exon-intron diagram of the *BBX* genes members was constructed according to their genomic and coding sequences (Additional file 1: Figure S2). The exon-intron distribution of all the studied *BBX* family members was investigated through GSDS online software. The range of a number of introns was from one to seventeen (*ZmBBX28*) in this study. However, we also identified some *BBX* members without of intron, they comprised only of the exon. For instance, *ZmBBX9*, *ZmBBX17*, *ZmBBX24*, and *ZmBBX33* genes have the only exon in maize. In rice, *OsBBX2* and *OsBBX25* were found without of intron. However, without of intron genes were not found in sorghum, stiff brome, and millet.

Furthermore, all the BBX proteins were run on MEME tool to investigate the motifs (Additional file 1: Figure S2). MEME analysis found a total of 10 motifs and was named 1-10. Based on width, Motif-7 was the largest motif, whereas next spots were held by motif-8 and motif-2 (Additional file 1: Table S2). We observed that motif-2 was present in 126 out of 131 *BBX* members, followed by the shortest motif, named motif-6 (115 *BBX* members). The longest motif (motif-7) was only found in 10 *BBX* members. Similarly, each motif-8 and 9 were found in 11 *BBX* members.

### Chromosomal Location, Multiple Alignments and Gene Duplication of *BBX* Genes

The chromosome location and annotation information of the *BBX* genes showed that *BBX* genes are unevenly distributed on the chromosomes in the genome of the studied species (Additional file 1: Figure S3). In maize, all the 36 *ZmBBX* genes were found to be distributed on the 10 chromosomes, except for chromosomes 8 (Additional file 1: Figure S3A). However, the number of *ZmBBX* genes varied widely on each chromosome. A high number of *ZmBBX* genes (7) was localized on chromosome no. 5, whereas 6, 5, 5, 4, 4, 3, 1 and 1 *ZmBBX* members were identified on chromosomes 4, 6, 9, 2, 10, 1, 3 and 7 in the maize genome, respectively. In rice, all 30 *OsBBX* genes are distributed on chromosomes 1–9: 8 *OsBBXs* are located on chromosome 6, 7 rice *BBX* members were detected on chromosomes 2, 3 *OsBBX* genes on each chromosome 4, 8 and 9, 2 *OsBBX* genes were found on chromosome 3, while 1 each on chromosomes 1, 5 and 7 (Additional file 1: Figure S3B). In sorghum, *SbBBX* genes were found to be distributed on all the chromosomes except chromosome 5: 8 *SbBBXs* were found on chromosome 4, 6 *SbBBXs* on chromosome 10, 3 *SbBBXs* were detected on chromosome 6, 2 *SbBBX* members are present on each 1, 2 and 7, while one each on chromosomes 3, 8 and 9 (Additional file 1: Figure S3C). All the *BdBBX* genes member are distributed on all chromosomes in stiff brome genome. A maximum number of *BdBBX* genes are localized on chromosome 1 (8 *BdBBXs*) and 3 (7 *BdBBXs*). Remaining *BdBBX* members are distributed as: 3 *BdBBXs* on chromosome 5, while 2 *BdBBX* genes are located on each chromosome 2 and 4. *SiBBX* genes were detected on all chromosome expect on chromosome 8 (Additional file 1: Figure S3D). The number of *BBX* genes on the chromosome is varied in millet genome. However, a high number of *SiBBX* (6) genes were observed on chromosome 1, whereas the lowest number of *SiBBX* genes (1) was found on chromosome 1. 4 and 3 *SiBBX* members are located on chromosome 4 and 7, respectively. 2 *SiBBX* genes were investigated on each chromosome 2 and 3 (Additional file 1: Figure S3E).

Multiple online databases including Pfam, SMART, Inter Pro Scan, Conserved Domain Database (CDD), NCBI, and Scan Prosite were used to identify the conserved domains of the *Poaceae* BBX proteins. The family-specific domains of BBX proteins including B-box1, B-box2, and CCT conserved domains, were aligned by DNAMAN software, and their logos were constructed via Web Logo online tool (Additional file 1: Figure S4). Previous studies investigated that the CCT domains comprised are the most conserved family specific domain among B-box1, B-box2 and CCT domains (Additional file 1: Figure S5a,b,c) [4, 28], and similar results were obtained for *Poaceae* BBX proteins. Previously, it was also postulated that B-box1 domain is the highly conserved domain than B-box2 domain and deletion event occur in the B-box2 domain. We also found that B-box1 was more conserved compared with B-box2 domain signifying that the deletion process could happen in B-box2 domains during evolution (Additional file 1: Figure S5a, b).

**Fig. 2:**
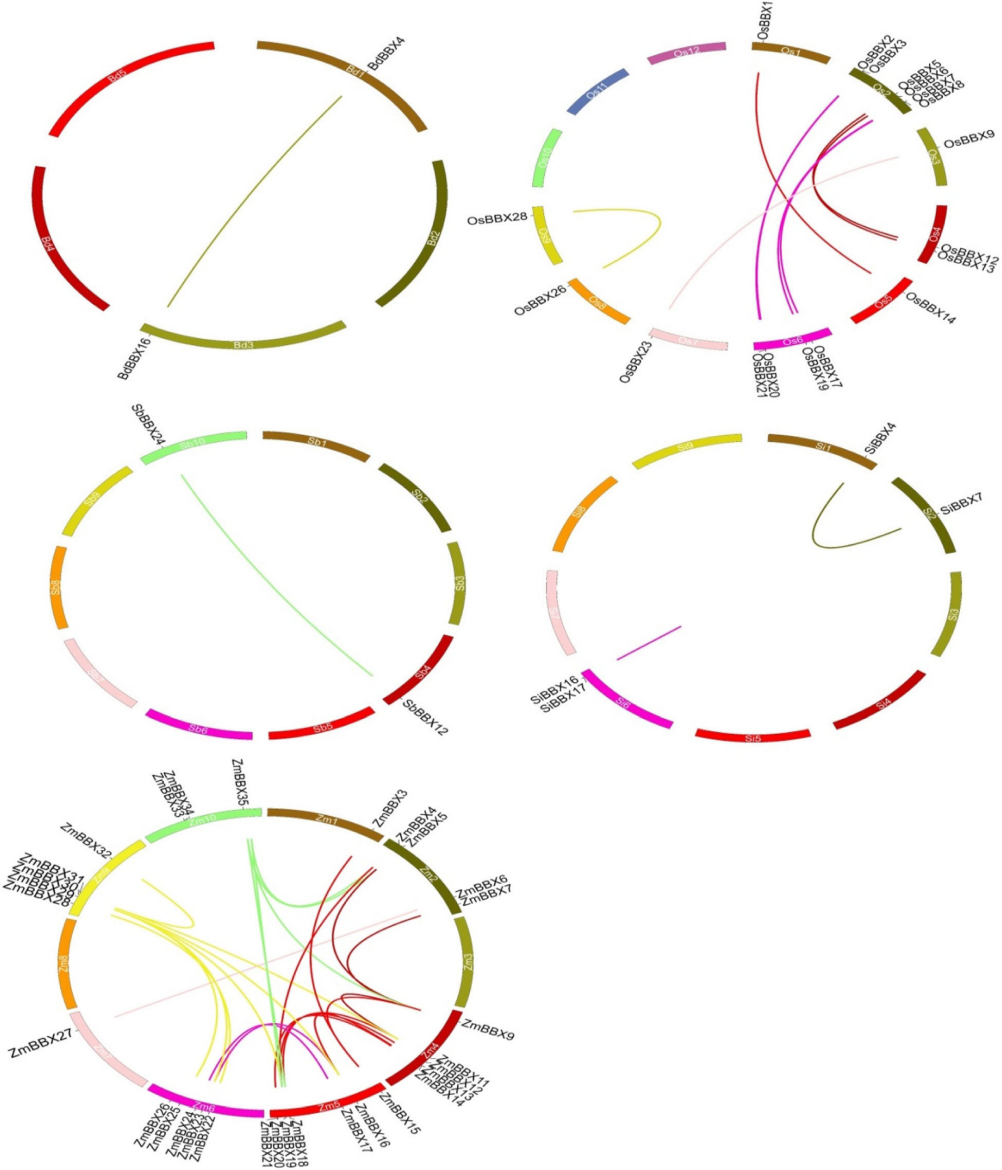
Synteny analysis of *BBX Poaceae* genes. Chromosomes of five *Poaceae* species are shown in different colors and in circular form. The approximate positions of the *BBX* genes are marked with a short black line on the circle. Colored curves denote the syntenic relationships between maize, rice, sorghum, stiff brome and millet

The duplication of individual genes, chromosomal segment, or of the entire genome itself are the major forces during the course of genome evolution in plants [29]. We identified the possibility of gene duplication in the *BBX* gene family in maize, rice, sorghum, stiff brome and millet (Fig. 2). A diagram constructed with the Circos program was used to draw the duplicated blocks in these plants genome. Both the segmental and tandem duplications were studied in this investigation. 25 *ZmBBX* pairs were located in the segmentally duplicated regions on different chromosomes in the maize genome. 9 *OsBBX* pairs of the duplicated region were found in the rice genome. Only one pair of the segmentally duplicated region was identified in each sorghum and stiff brome genome, whereas two pairs of the duplicated region of *BBX* genes were located on the chromosome in millet genome. However, no tandem duplication was observed among the *BBX* family members in the studied plants. The results indicated that only segmental duplication may take part in the evolution of *BBX* genes in maize, rice, sorghum, stiff brome, and millet.

### Developmental and Tissue-Specific Expression Profiles of Rice *BBX* Genes

**Fig. 3:**
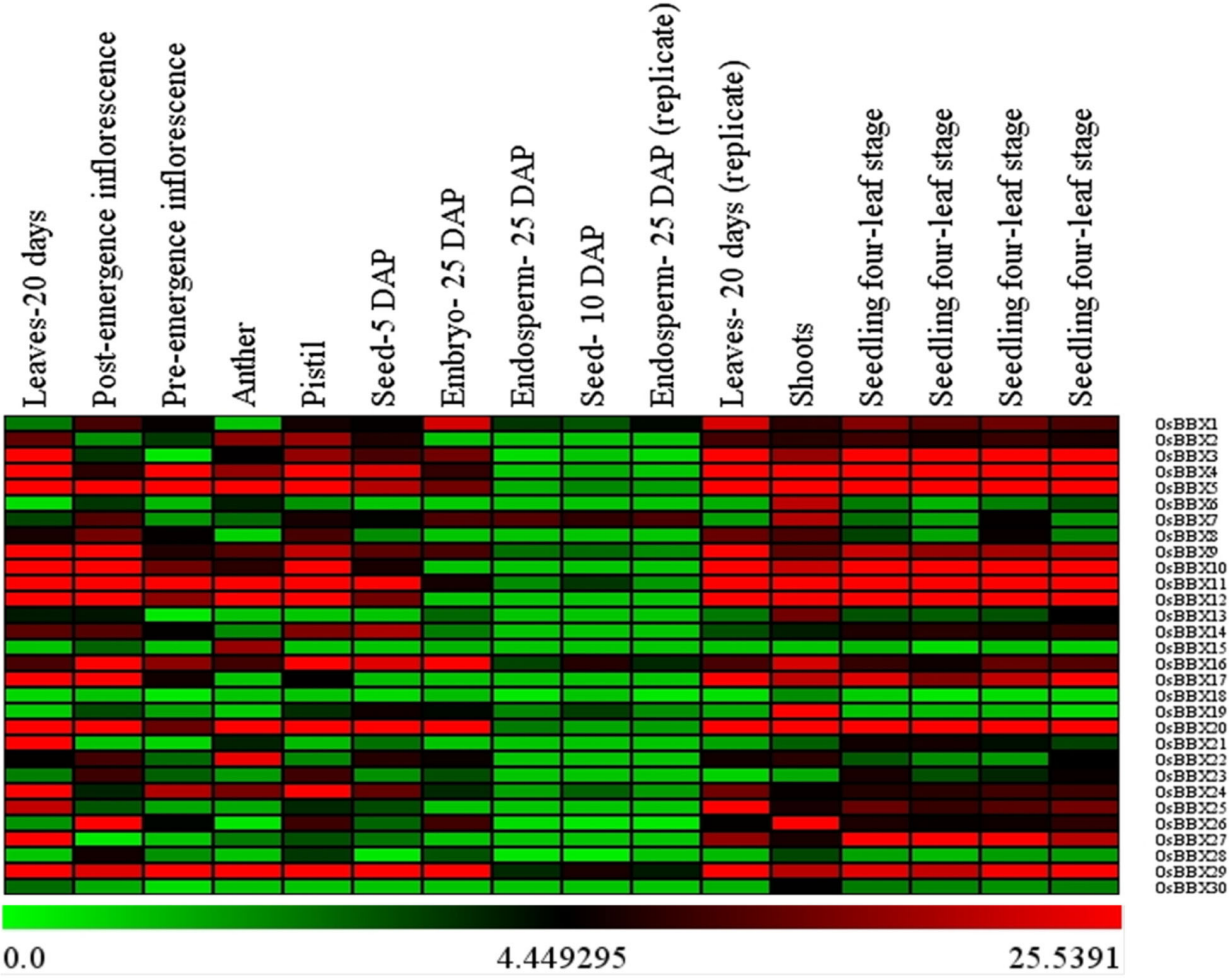
The expression profiles obtained from the ArrayExpress data, dsiplaying diverse expression levels of apple *BBX* genes in different tissues and organs. Relative transcript level of *BBX* genes members based on ArrayExpress data were presented as heat maps from green to red reflecting relative signal values; where dark green boxes represent stronger down-regulated expression and dark red boxes represents stronger up-regulation

We examined the different developmental stages/tissues to study the biological roles of *BBX* genes in the plant growth and development, based on a set of microarray data obtained from Genevestigator v3 and quantitative real-time polymerase chain reaction (qRT-PCR). The expression data from the microarray analysis of rice *BBXs* are presented in the form of a heat map, from blue to pink reflecting the percentage expression (Fig. 3). Nine tissues including seedling, shoot, leaves, seed, endosperm, embryo, anther, pistil, pre and post-emergence inflorescences, were analyzed. The 30 candidates of rice *BBX* genes displayed quite a similar expression profile among the tested tissues (Fig. 3). Eight members of rice *BBX* (*OsBBX4*, *OsBBX5*, *OsBBX9*, *OsBBX10*, *OsBBX11*, *OsBBX12*, *OsBBX20,* and *OsBBX29*) were highly expressed in seedling, shoot, leaves, seed-5 DAP, pistil, anther, pre and post-emergence inflorescences. No expression was detected for all the members of *BBX* genes in endosperm and seed-10 DAP except for *OsBBX7*, *OsBBX16* and *OsBBX29*; however, we found 17 *BBX* genes members (*OsBBX1*, *OsBBX2*, *OsBBX3*, *OsBBX4*, *OsBBX5*, *OsBBX7*, *OsBBX9*, *OsBBX10*, *OsBBX11*, *OsBBX12*, *OsBBX14*, *OsBBX16*, *OsBBX19*, *OsBBX20*, *OsBBX22*, *OsBBX24* and *OsBBX29*) with high transcripts in seed-5 DAP. No or extremely low transcript level was detected for *OsBBX6*, *OsBBX18*, *OsBBX28,* and *OsBBX30* among all the studied tissues. Moreover, we observed the expression profile of two *BBX* genes, namely *OsBBX16* and *OsBBX29*, among all the tissues apart from endosperm-25 DAP, seed-10 DAP and endosperm-25 DAP (replicate). This investigation found that all the *BBX* genes were expressed in the shoot except *OsBBX15*, *OsBBX18*, *OsBBX21*, *OsBBX23,* and *OsBBX28.*

**Fig. 4:**
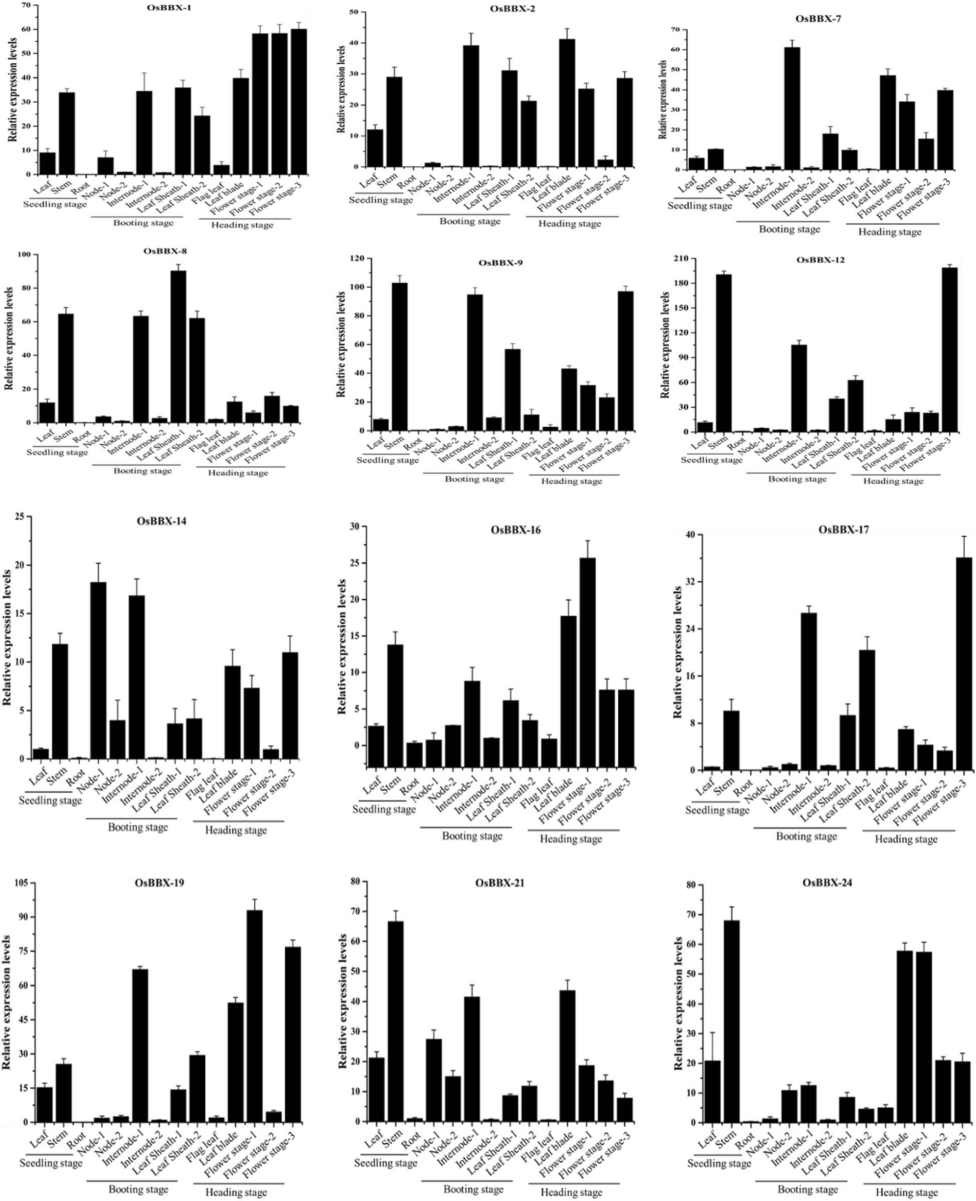
Expression profile of the *OsBBX* genes in tested tissues. The graphs indicate tissue specific expression level in rice plant. The samples were collected in different developmental stages, and were analyzed through qRT-PCR. The x-axis indicates the tissues. The y-axis shows the relative expression level of each tissue. The error bars indicate the standard deviations of the three independent qRT-PCR biological replicates

Furthermore, we performed qRT-PCR of the 12 rice *BBX* members (*OsBBX1*, *OsBBX2*, *OsBBX7*, *OsBBX8*, *OsBBX9*, *OsBBX12*, *OsBBX14*, *OsBBX16*, *OsBBX17*, *OsBBX19*, *OsBBX21* and *OsBBX24*) to find out the expression profiles among 14 different tissues (Fig. 4). The tissues were collected at three different stages: 1) seedling stage including leaf, stem and root; 2) booting stage consisted node-1, node-2, internode-1, internode-2, leaf sheath-1, and leaf-sheath-2; 3) heading stage including flag leaf, leaf blade, flower stage-1, flower stage-2 and flower stage-3. The transcript levels of all the studied *BBX* genes were high in the stem, internode-1, and flower stage-3 tissues. All the 12 *BBX* members showed low transcription in the root, flag leaf, and internode-2 tissues. No high expression was detected for the all the *BBX* genes in node-2 except for *OsBBX14*, *OsBBX16*, *OsBBX21,* and *OsBBX24*. Low transcript level was observed for *OsBBX17* gene in leaf, whereas high transcript level was detected for the remaining *BBX* members. The expression profile of all the *BBX* genes was almost similar in node-2 and internode-2. High expression profile was found for *OsBBX1*, *OsBBX2*, *OsBBX7*, *OsBBX8*, *OsBBX12* and *OsBBX17* in leaf sheath-1 and leaf sheath-2, while the rest of *BBX* members showed low expression profile in these two tissues. In leaf blade and flowering stage-1, the expression profile of all *OsBBX* genes was maximum except *OsBBX8*, *OsBBX12,* and *OsBBX17*. The transcription rate of all *BBX* members was high in flowering stage-2 excluding *OsBBX14*, *OsBBX17*, and *OsBBX19*. Overall, we noted that the transcript level of most rice *BBX* genes was high in the heading stage, followed by booting and seedling stage based on the three stages. The present study found the expression profile (low or high) of *OsBBXs* in almost all the tested tissues. These findings indicated the multiple roles of *BBX* gene family in the development and growth of rice.

### Inducible Expression Analysis of Rice *BBX* Genes under Abiotic stresses and hormonal applications

**Fig. 5:**
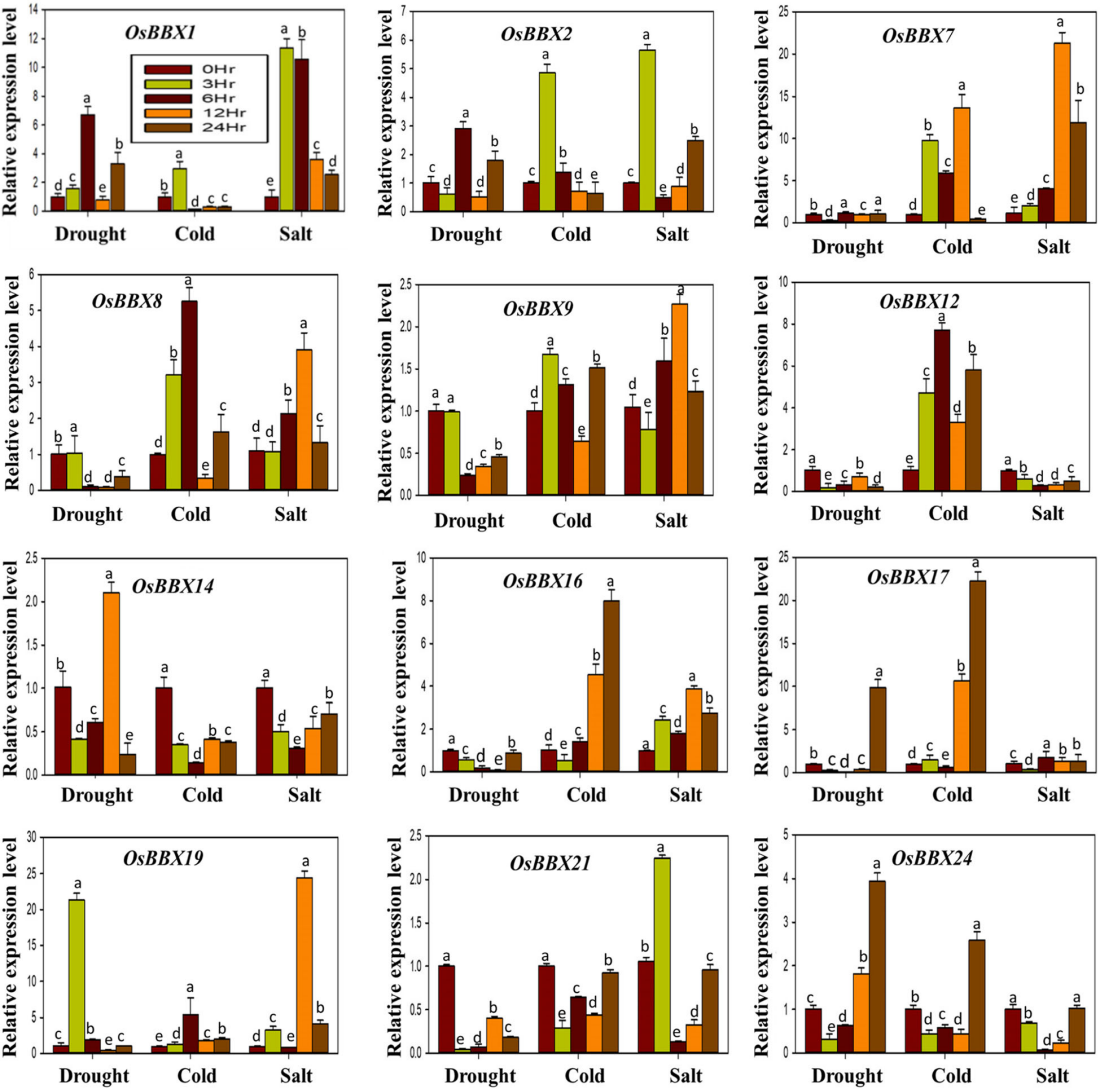
Inducible expression profile of rice *BBX* gene family members in response to abiotic stresses. The x-axis indicates the treatment. The y-axis shows the relative expression level of each treatment compared to control (0h). The error bars indicate the standard deviations of the three independent qRT-PCR biological replicates. Small letters (a–e) represent significant difference (*p* < 0.05)

Gene expression analysis can provide essential clues for gene function; therefore, we carried out qRT-PCR to investigate the transcript levels of the rice *BBX* genes under different abiotic stresses, including drought, cold and salt. Describing the expression profiles of all rice *BBX* genes was exhaustively difficult; therefore, twelve *BBX* members (*OsBBX1, OsBBX2, OsBBX7, OsBBX8, OsBBX9, OsBBX12, OsBBX14, OsBBX16, OsBBX17, OsBBX19, OsBBX21,* and *OsBBX24*) of rice *BBX* gene family were assessed (Fig. 5). More than two-fold difference in transcript levels was considered to be the true difference for the genes under treatments. We found that the transcript levels of *OsBBX7*, *OsBBX8*, *OsBBX9*, *OsBBX12*, *OsBBX16,* and *OsBBX21* were down-regulated, whereas the remaining six *BBX* members were up-regulated at least at one (*OsBBX14*, *OsBBX17*, and *OsBBX19*) or two-time points (*OsBBX1*, *OsBBX2*, and *OsBBX24*) under drought stress. Under cold stress, the expression profile of only one *BBX* gene (*OsBBX12*) was high at all the tested time points compared to 0 hr sample (control), whereas the expression profile of *OsBBX14* and *OsBBX21* was down-regulated. The expression of *OsBBX1* and *OsBBX2* and *OsBBX19* was high at 3 hr and 6 hr time points, respectively, while the other six *BBX* members were up-regulated at two or three time points under cold stress. Similarly, the transcript profile of *OsBBX1*, *OsBBX7*, *OsBBX8,* and *OsBBX16* was high at all the time points under salt stress. Moreover, some *BBX* members (*OsBBX12*, *OsBBX14*, *OsBBX17,* and *OsBBX24*) were down-regulated, while the rest of the four *BBX* genes up and down-regulated at different time points under salt stress. Altogether, we observed that transcript of most rice *BBX* members was significantly affected under salt and cold stresses; in addition, we also noticed that the *BBX* members were also up and down-regulated at some time points under drought conditions. All these results indicate the involvement of *BBX* gene family in plant growth and development and their response against multivariate stresses.

**Fig. 6:**
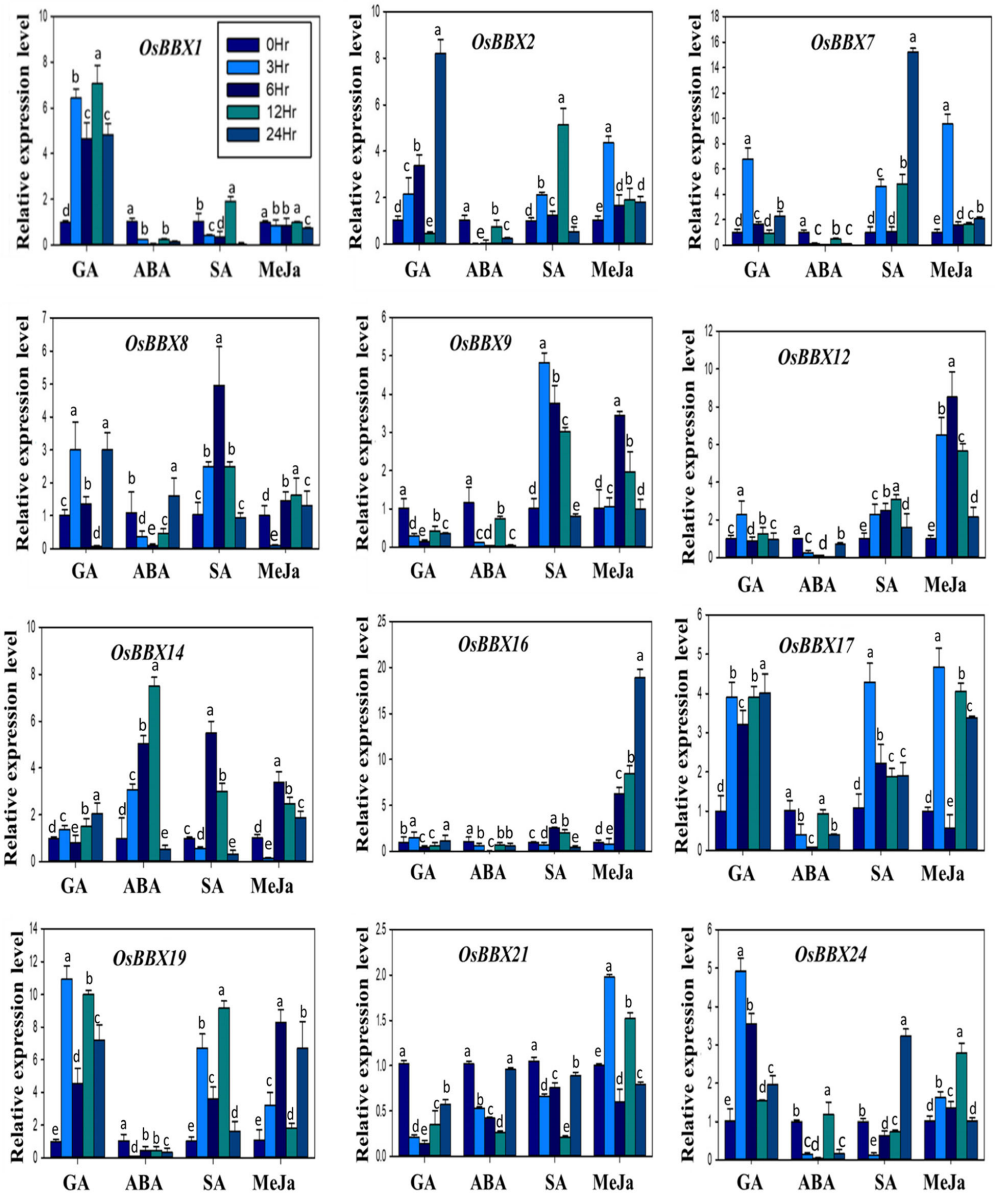
Inducible expression profile of rice *BBX* gene family under exogenous hormones. The x-axis indicates the treatment. The y-axis shows the relative expression level of each treatment compared to control (0h). The error bars indicate the standard deviations of the three independent qRT-PCR biological replicates. Small letters (a–e) represent significant difference (*p* < 0.05)

qRT-PCR was also used to analyze the transcript patterns of all *BBX* genes under GA, ABA, SA, and MeJA hormones applications, to reveal the effects of various hormones on the expression of *BBX* gene family members in rice (Fig. 6). We noticed that the expression levels of *OsBBX1*, *OsBBX17*, *OsBBX19,* and *OsBBX24* were promoted in response to exogenous GA treatment at all the time points, whereas the transcripts of *OsBBX9* and *OsBBX21* were down-regulated. Furthermore, *OsBBX2*, *OsBBX7,* and *OsBBX8* were up-regulated at 3, 6 and 24 hr. We also found low transcripts for some *BBX* members including *OsBBX12*, *OsBBX14* and *OsBBX16* genes under GA treatment. In contrast, the expression levels of all rice *BBX* gene members were very low excluding *OsBBX14* under ABA treatment. Moreover, the transcript levels of *OsBBX12*, *OsBBX17,* and *OsBBX19* were up-regulated at all the time points under SA hormone, whereas *OsBBX21* was down-regulated. We found some genes members, namely *OsBBX2* and *OsBBX9*, with high expression profiles till 12 hr post-treatment and their expression was suddenly declined at the 24 hr time point. The expression of *OsBBX1* was increased at only one time point (12 hr). We also observed a maximum number of *BBX* members shown up-regulation in expression at 3, 6 and 12 hr time points under SA treatment. Under MeJA hormones, most rice *BBX* was up-regulated at least one or two time points, however, *OsBBX2* and *OsBBX12* were up-regulated at all the time points. Low transcript level was detected for *OsBBX1* and *OsBBX8* at all the time points in response to exogenous MeJA treatment. Overall, the expressions of rice *BBX* genes members were highly affected by exogenous GA, SA and MeJA hormones. Additionally, the transcripts of rice *BBX* members were also changed by exogenous ABA treatment at a few time points. Thus, the results reveal that in response to signaling molecules the *BBX* genes members underwent clear variations in transcript level suggesting their hormone-induced differential responses in rice.

### Expression Profiles of rice *BBX* genes under metals treatments

**Fig. 7:**
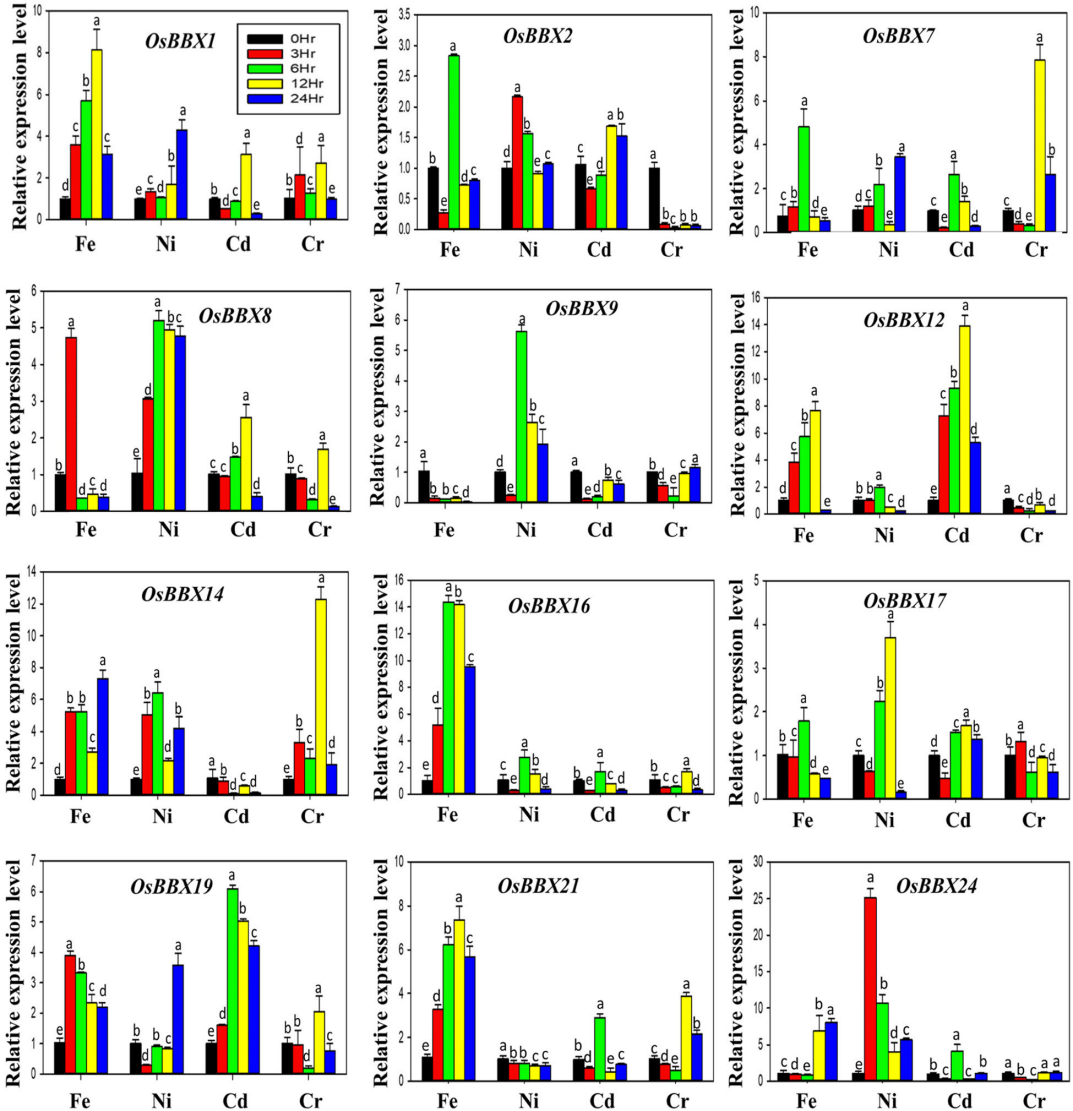
Inducible expression profile of rice *BBX* gene family members in response to heavy metals. (A), Chromium (Cr); (B), Cadmium (Cd); (C), Nickel (Ni)*;* (D), Iron (Fe), respectively. The x-axis indicates the treatment. The y-axis shows the relative expression level of each treatment compared to control (0h). The error bars indicate the standard deviations of the three independent qRT-PCR biological replicates. Small letters (a–e) represent significant difference (*p* < 0.05)

Two-week-old rice plants were exposed to four different metals stressors such as Cr, Cd, Ni, and Fe, to insight the transcriptional regulation and expression profiles of rice *BBX* genes, and the possible involvement of heavy metal stresses (Fig. 7). The temporal induction of rice *BBX* genes members at the transcriptional level at a various time point were evaluated through qRT-PCR. We found that the transcript profiles of *OsBBX1*, *OsBBX7*, *OsBBX8*, *OsBBX17,* and *OsBBX19* were affected by all the four metals including Cr, Cd, Ni and Fe metals at some time points. The expression profiles of *OsBBX2* and *OsBBX14* genes were up-regulated under all four metal stresses apart from Cr and Cd, respectively. The transcription patterns of *OsBBX9* had shown obvious changes in the expression level under Ni stress; likewise, *OsBBX16* and *OsBBX21* were up-regulated by Fe stress while the response of these genes to other metals such as Ni, Cr, and Cd was very low. Similarly, the expression level of *OsBBX24* gene was high at 3 and 6 hr under Ni metal, while low transcript was noticed under other three metal treatments. For *OsBBX12*, low transcript level was observed under Ni and Cr metal, however, the expression was up-regulated under Fe and Cd metal stresses. Based on time points, we noticed that most rice *BBX* members were up-regulated at 12 hr time point followed by 6, 3 and 24 hr, respectively. Furthermore, based on metals, this study observed the expression of almost all the *BBX* members shown up-regulation at least at one time point under Fe and Ni metals excluding *OsBBX9* and *OsBBX21* genes, respectively. In response to Cr and Cd, rice *BBX* genes showed a low level of expression apart from *OsBBX8*, *OsBBX12,* and *OsBBX19* and *OsBBX7* and *OsBBX14*, respectively. Overall, the studied *BBX* members showed high expression profiles in Fe and Ni compared with Cr and Cd metals. The unique inducible expression patterns of the *BBX* gene family members under metal stresses may indicate the role of *BBX* genes family in response to heavy metals. However, further studies are required to investigate deeply the particular behavior role of *BBX* gene family in plant multivariate stresses.

## Discussion

The gene clustering and evolutionary relationship mostly change due to domain shuffling and low sequence identity among the homologs proteins. Therefore, the rearrangement of domain composition, exon shuffling and gene duplication may lead to the expansion of gene families in plants during evolutionary processes [29, 30]. Subsequently, the duplicated gene may promote functional variations, and possibly expand the functional characteristics of genes [31, 32]. Furthermore, single gene duplication might be the main cause leading to the expansion of gene families in plants.

### Identification and Evolution history of *BBX* family members in various plants

*BBX* gene family has been got more attention from the scientific community in the past couple of years. The genome-wide identification analysis of *BBX* genes has been already investigated in rice [14] and other important plants such as *Arabidopsis*, tomato, potato, pear and apple [1, 14, 33–36]. In this study, we also reported the genome-wide identification of *BBX* genes in five *Poaceae* species (maize, rice, sorghum, millet, and stiff brome), and their expression analysis under abiotic (cold, drought and salt), hormones (GA, ABA, SA and MeJA) and metal stresses (Cr, Cd, Ni and Fe) in rice. Based on our results, we found a total of 131 *BBX* genes in the five *Poaceae* species genomes including 36 from maize, 30 from rice, 24 from sorghum, 22 from stiff brome and 19 from millet (Table 1). The previous study also found a similar number of *BBX* genes in the rice genome [14]. The number of *BBX* gene family members is pretty consistent among different crop plants, such as 30, 32, 29 and 30 *BBX* genes members were already identified in rice, *Arabidopsis*, tomato, and potato, respectively [1, 14, 33, 36]. The difference in the number of *BBX* genes among the crops plants is very less. However, a total of 67 *BBX* genes in apple [35]. The difference in the number of *BBX* genes between tree and crop plants may due to the large and heterozygous genome of apple. Furthermore, we also found less number of *BBX* members in two species of *Poaceae* family, 22 from stiff brome and 19 from millet. The difference may due to the genome of these two species are not fully sequenced or may small and simple genome.

Previous studies identified 4 different types of *BBX* proteins based on domain organization in tomato and *Arabidopsis* [1, 36]. We also found 4 different types of BBXs (Table 2), BBXs with only one B-box domain, BBXs with two B-boxes domains, BBXs with one B-box and additional CCT domains and BBXs with two B-boxes and additional CCT domains. However, we detected a small difference in the composition of a different class of BBXs in different species. The numbers of *BBX* with only one B-box domain, two tandem B-boxes, BOX1 plus CCT, two tandem B-boxes plus the CCT domain were 7, 8, 4, and 13, and 6, 10, 5, and 8 in *Arabidopsis* and tomato, respectively, however this arrangement was 3, 10, 10 and 7 in rice, 4, 17, 10 and 5 in maize, 2, 8, 9 and 5 in sorghum, 1, 10, 7 and 3 in stiff brome, and 1, 8, 5 and 5 in millet. The results indicate that *BBX* gene family may share conserved gene architecture and domain organization in plants during the evolution process.

The *Arabidopsis BBX* was clearly divided into five clusters on the basis of different conserved domains combinations [1]. Two B-boxes plus additional CCT domains containing *BBX* (*AtBBX1*-*AtBBX13*) were found in group-1 and 2; one B-box plus CCT domain containing genes (*AtBBX14*-17) were clustered into group-3, *BBX* genes containing two B-boxes (*AtBBX18*-25) and one B-box domains (*AtBBX26*-32) were observed in clade-4 and 5 in Arabidopsis, respectively [1]. Whereas, in five *Poaceae* species, maximum number of one and two B-boxes and additional CCT conserved domains containing *BBX* genes members were cluster together into subfamily I, II and III (Fig. 1), *BBX* genes possessing one B-box domain were detected in subfamily II, IV and V, whereas two B-boxes containing *BBX* genes were observed in subfamily IV and V in this study. The classification of *Poaceae BBX* members based on conserved domain was relatively difficult. The reason behind uneven distribution may due to a large number of genes or the small difference in the domain organization in the plant species. For instance, we noticed that 7 *BBX* genes possessed only one B-box domain, 8 *BBX* members had two B-boxes domain, 4 *BBX* members contained one B-box and additional CCT domain and 13 *BBX* genes were found with two B-boxes and additional CCT domains in *Arabidopsis* [1]. In contrary, 3 *BBX* possessed only one B-box domain, 10 *BBX* found having two B-boxes domains, one B-box and additional CCT domain were observed in 10 *BBX* members and 7 *BBX* genes comprised of two B-boxes and additional CCT domains in rice (Table 2). Similar differences were also observed for B-box genes in other four studied *Poaceae* species. However, we also noted that the gene structure and functional characteristic of *BBX* genes within the subfamily was quite similar. Thus, it is assumed that *BBX* members share a similar gene structure and functional characteristic within the same subfamily during the evolutionary relationship. Previously, it also has been reported that FRO gene family members in rice shared similar gene structure and functional characteristic during evolution in rice [37].

Moreover, It has been already reported that CCT is the highly conserved domain [29, 38]. The alignment of B-box1, B-box2 and CCT domain also indicated that the CCT domain was highly conserved compared with B-box1 and B-box2 domain (Additional file 1: Figure S5a, b, c). However, a theory has been proposed that a deletion process occurs during the evaluation that leads to making another class of *BBX* genes, containing only one B-box domain [3]. After detail sequence alignment of two B-box domains (B-box1 and B-box2) revealed that B-box1 domain was highly conserved compared with B-box2 in rice *BBX* (Additional file 1: Figure S5a, b), thus, it’s postulated that deletion process could occur in the B-box2 domain and give birth to the B-box1 domain.

Large-scale duplication and tandem duplication processes are vital for the amplification of gene family members in the genome during the evolution [39]. In this study, both the tandem and segmental duplication events were analyzed to study the evaluation of the *BBX* genes in *Poaceae*. We found only segmental duplication in the *BBX* genes (Fig. 2) indicating that segmental duplication events took part in the expansion of the *BBX* gene family in *Poaceae*.

### Tissue-Specific gene expression profiles reveal the diverse roles of *BBX* gene family in plant growth and development

The specific gene family members have common genes expression profile features in plants. This may coordinate and/or differ in the functional interaction of the family members. It was previously reported that BBX proteins control the diverse functions of the plant, such as photomorphogenesis, flowering and shade avoidance [40, 41]. In *Arabidopsis*, the overexpression of a *BBX* gene (BBX6, COL5) promotes early flowering [42], and the overexpression COL9 (BBX7) delay the flowering under SD (short day) condition [43]. *BBX* homologous genes which contribute to different biological processes with obvious tissue specificity in gene expression have been functionally characterized in maize [44]. The members of *BBX* gene family also showed diverse expression in all the tested tissues in tomato [36]. Similarly, in potato maximum number of *BBX* family members was detected with distinct expression pattern among the tested organs [33]. Likewise, we investigated the expression of *BBX* family in 14 different tissues and the samples were collected at three different stages, seedling stage root, booting stage and heading stage (Fig. 4). We found that the expression of almost all the *BBX* members was high in all the tested samples apart from roots. Furthermore, we also noticed that the transcript levels of the studied *BBX* members were high in the heading stage. Moreover, the database searching found that *BBX* gene more expressed seedling, leaf, shoot and flowering-related tissues (Fig. 3). Thus, the database searching and functional prediction of *BBX* gene family members in various tissues and different developmental stages demonstrate that *BBX* gene family might play vital roles in plant growth, and some *BBX* genes members might have a unique function in specific developmental stages.

### Pronounced but differentiated inducible expression patterns under a number of environmental, hormonal and metal stresses imply the vital contributions of *BBX* gene members to multivariate stress tolerance

Various adverse environmental aspects such as ion toxicity, salinity, drought, extreme temperatures negatively disturb plant growth and development [45–47]. Among them, several abiotic stresses cause general or specific effects on growth and development and changes at the transcriptional level in plants [48–50]. Here, we detected that rice *BBX* genes are sensitive to a set of abiotic stresses, and their transcriptional expressions were greatly altered by salt, cold, drought, GA, SA, MeJA, ABA and metals stress treatments, displaying their contribution in responses to multiple stresses in rice. Several investigations have proposed that *BBX* genes are important for the photoperiodic regulation of flowering, seedling photomorphogenesis, shade avoidance, and responses to biotic and abiotic stresses. It has been also stated that the salt tolerance protein STO (AtBBX24) enhances the growth of root under a high salinity condition in *Arabidopsis* [15] and the salt tolerant activities was also triggered in yeast cells [16]. *AtBBX18* acts as negative regulator both in photomorphogenesis and thermotolerance in *Arabidopsis* [12]. Furthermore, *BBX18* negatively regulates the expression of heat-responsive genes such as *DGD1*, *Hsp70*, *Hsp101*, and *APX2*, thereby reducing germination and seedling survival after a heat treatment [12]. In *Chrysanthemum, CmBBX24* performs a dual function, delaying flowering and also increasing cold or drought tolerance in the plant [19]. Moreover, some studied found that BBX proteins also involve in hormones signaling. A recent investigation found that the expression pattern of *BBX* genes was altered in response to ABA and cyclic ADP-ribose (cADPR) temperatures [6, 7]. The involvement of *BBX* genes in the COP/HY5 signaling pathway indicates that *BBX18* may work as an integrator of both GA and COP1/HY5 pathways [13]. Based on the previous studies, we evaluated the expression of *OsBBX* genes in response to numerous abiotic and hormonal stresses and found that the most rice *BBX* members show high expression levels under abiotic stresses (Fig. 5). The expression patterns of *OsBBX1*, *OsBBX2,* and *OsBBX19* genes were affected by all the three used abiotic stresses including drought, salt and cold stresses. *OsBBX7*, *OsBBX8,* and *OsBBX16* genes showed high expression under salt and cold conditions, whereas *OsBBX17* and *OsBBX24* genes were up-regulated in response to drought and cold. In addition, we found that most rice *BBX* genes were up-regulated under the cold and salt condition, while, less transcript level was observed for most rice *BBX* genes in response to drought. The members of rice *BBX* gene family also showed maximum expression levels in response to different hormones (Fig. 6). The expression of *OsBBX2*, *OsBBX7*, *OsBBX17*, *OsBBX19,* and *OsBBX24* genes were strongly triggered in response to GA, SA and MeJa hormones. Similarly, *OsBBX1* and *OsBBX16* genes displayed high expression under GA and MeJa hormones, respectively. Moreover, the transcript levels of *OsBBX8* and *OsBBX14* were promoted under GA, ABA, SA and MeJa hormones. Although most rice *BBX* genes were up-regulated at different points under GA, SA and MeJA hormones, the transcripts of the *BBX* gene family were less effected by ABA. Furthermore, the transcript levels of most *BBX* members were significantly stimulated by heavy metal stresses even though somewhat unique responses occurred for some members under certain metals (Fig. 7). For example, the transcript profiles of *OsBBX1*, *OsBBX7*, *OsBBX8*, *OsBBX17*, and *OsBBX19* members were greatly affected by Fe, Ni, Cr, and Cd metals, however, the transcription activity of *OsBBX24* was significantly changed in response to all the applied metals apart from Cr metal. Similarly, the transcript profile of *OsBBX14* was enhanced in response to all used metals except Cd metal. Furthermore, we also found some *BBX* genes which showed high expression profile in response to only one metal, for instance, *OsBBX9* was highly expressed under Ni metal. Overall, the results obtained here suggest that *BBX* gene family may perform several functions in plant growth and development and in response to abiotic, metal stresses and hormonal applications although their exact role remains unclear. Further experiments need to be done to investigate the exact role of *BBX* gene family in plant growth and development.

## Conclusions

Over a long evolutionary relationship of plants, *BBX* genes had shown consistency in their common characteristics and functional behavior. In this context, the differential expression patterns of *BBX* genes in *Poaceae* plants play a vital role in the plant growth regulation. The regulatory mechanism and transcriptional variation of *BBX* genes are highly responsive to external factors, thus, the multivariate stresses and hormonal application substantially triggered the up-regulation of the differentially expressed genes, thereby participating the beneficial allocation and potential role of these genes in plants. We suggest that the specific role of particular *BBX* gene should be a target for defining the stress response, functional divergence and possible crosstalk in plants such as rice.

## Additional file

Additional file 1: Supplementary Figures and Supplementary tables. (DOCX 5100 kb)

## Abbreviations

GA: Gibberellic acid
ABA: abscisic acid
MeJA: methyl jasmonate
SA: salicylic acid
Ni: Nickle
Fe: Iron
Cd: Cadmium
Cr: Chromium
