# Supplementary material for: Characterization of B-BOX gene family and their expression profiles under hormonal, abiotic and metal stresses in Poaceae plants
Source: BMC Genomics. 2019 Jan 9;20:27. doi: 10.1186/s12864-018-5336-z (PMC6327500; doi:10.1186/s12864-018-5336-z)

**Table S1:** The detail information about physiochemical characteristics of BBX Gene family in five *Poaceae* species.

| **Name** | **MW** | **PI** | **-Ve CR** | **+Ve CR** | **N.A** | **Ii** | **AI** | **GRAVY** | **Composition %** |
| --- | --- | --- | --- | --- | --- | --- | --- | --- | --- |
| **OsBBX1** | 36.67 | 5.83 | 37 | 28 | 5024 | 57.37 | 62.18 | -0.298 | (A) 13.6, (S) 11.3, (G) 9.3 |
| **OsBBX2** | 22.84 | 10.50 | 22 | 34 | 3181 | 76.62 | 75.45 | -0.139 | (A) 22.5, (R) 14.0, (S) 10.4 |
| **OsBBX3** | 33.87 | 5.94 | 38 | 32 | 4636 | 48.56 | 64.83 | -0.175 | (A) 18.7, (G) 12.7, (P, S) 6.9 |
| **OsBBX4** | 29.43 | 4.93 | 40 | 21 | 3991 | 41.76 | 69.34 | -0.330 | (A) 15.9, (D) 8.1, (P, L) 7.4 |
| **OsBBX5** | 34.93 | 5.11 | 50 | 37 | 4797 | 44.02 | 67.35 | -0.242 | (A) 21.4, (D) 30, (S, R) 8.1, |
| **OsBBX6** | 28.40 | 5.36 | 30 | 22 | 3845 | 66.62 | 63.35 | -0.233 | (A 16%, S 13.0%, L 6.7 |
| **OsBBX7** | 44.20 | 5.10 | 57 | 43 | 6033 | 49.89 | 58.55 | -0.581 | (S) 11.8, (A, D) 8.6, (G) 6.9 |
| **OsBBX8** | 38.50 | 4.77 | 59 | 38 | 5252 | 50.84 | 63.05 | -0.503 | (A) 11.0, (S) 8.9, (E) 8.6 |
| **OsBBX9** | 43.18 | 5.36 | 55 | 40 | 5936 | 46.54 | 60.40 | -0.575 | (A) 11.9, (G) 8.9, (E) 7.9 |
| **OsBBX10** | 45.51 | 6.21 | 65 | 60 | 6351 | 55.84 | 62.54 | -0.617 | (A) 15.0, (P) 10.7, ® 10.5 |
| **OsBBX11** | 19.68 | 4.89 | 24 | 16 | 2698 | 51.46 | 72.23 | -0.353 | (A, P) 12.0, (S) 10.9, (D) 8.2 |
| **OsBBX12** | 34.23 | 5.04 | 53 | 38 | 4723 | 35.86 | 62.46 | -0.380 | (A) 20.7, (D) 10.5, (R) 7.8 |
| **OsBBX13** | 26.80 | 5.12 | 35 | 25 | 3661 | 64.04 | 67.28 | -0.321 | (A) 13.6, (S) 11.6, (E) 8.0 |
| **OsBBX14** | 39.78 | 5.48 | 40 | 28 | 5392 | 74.30 | 54.63 | -0.417 | (P) 13.5, (A) 11.6, (S) 10.3 |
| **OsBBX15** | 24.46 | 9.09 | 16 | 26 | 3314 | 58.01 | 63.23 | -0.402 | (A) 13.0, (L) 9.0, (P) 8.5 |
| **OsBBX16** | 38.67 | 5.26 | 39 | 27 | 5271 | 50.57 | 69.42 | -0.348 | (A) 10.0, (S) 11.7, (L) 7.8 |
| **OsBBX17** | 51.77 | 5.42 | 71 | 60 | 7146 | 56.08 | 65.72 | -0.498 | (A) 15.0, (R) 8.9, (D) 7.7 |
| **OsBBX18** | 43.41 | 4.84 | 53 | 36 | 5862 | 42.05 | 63.04 | -0.542 | (A) 11.4, (S) 9.1, (N, V) 7.6 |
| **OsBBX19** | 43.87 | 5.08 | 54 | 42 | 6014 | 49.11 | 64.14 | -0.448 | (A) 8.8, (D) 8.3, (S) 14.2 |
| **OsBBX20** | 9.51 | 9.75 | 14 | 19 | 1299 | 38.19 | 44.81 | -1.104 | (R) 16.0, (A) 14.8, (D) 11.1 |
| **OsBBX21** | 24.93 | 9.40 | 26 | 35 | 3427 | 62.51 | 73.69 | -0.125 | (A) 27.4, (R) 13.3, (G) 8.7 |
| **OsBBX22** | 31.66 | 5.08 | 42 | 28 | 4390 | 56.17 | 73.08 | -0.149 | (A) 18.5, (S) 9.4, (D) 8.8 |
| **OsBBX23** | 40.86 | 5.04 | 56 | 42 | 5547 | 53.37 | 68.45 | -0.407 | (A) 12.6, (S) 9.7, (G) 8.9 |
| **OsBBX24** | 28.19 | 4.75 | 47 | 29 | 3784 | 58.08 | 45.62 | -0.718 | (G) 19.9, (A) 13.2, (E) 11.4 |
| **OsBBX25** | 17.37 | 4.00 | 18 | 5 | 2282 | 41.63 | 57.94 | 0.183 | (A) 27.8, (S) 13.3, (G) 11.1 |
| **OsBBX26** | 51.77 | 6.21 | 62 | 57 | 7124 | 47.24 | 61.97 | -0.547 | (A) 13.9, (G) 8.6, (S) 8.4 |
| **OsBBX27** | 36.02 | 5.37 | 48 | 39 | 4944 | 59.40 | 58.63 | -0.469 | (S) 11.6, (A) 11.0, (D) 8.7 |
| **OsBBX28** | 46.55 | 7.15 | 57 | 57 | 6350 | 47.71 | 59.07 | -0.588 | (A) 14.6, (G) 10.2, (R) 10.0 |
| **OsBBX29** | 23.18 | 5.90 | 31 | 25 | 3178 | 46.44 | 60.47 | -0.685 | (D) 10.0, (V) 9.0, (A) 6.6 |
| **OsBBX30** | 21.79 | 4.99 | 27 | 14 | 2928 | 47.73 | 69.48 | -0.137 | (A) 18.1, (S) 10.5, (D) 8.6 |
| **ZmBBX1** | 42.32 | 5.26 | 59 | 45 | 5831 | 52.63 | 57.68 | -0.674 | (A)12.6, (G)9.1, (P)8.1 |
| **ZmBBX2** | 51.29 | 5.88 | 62 | 53 | 7048 | 44.63 | 66.83 | -0.396 | (A) 14.9, (S) 9.6, D (8.2) |
| **ZmBBX3** | 51.38 | 5.96 | 73 | 66 | 7200 | 58.42 | 65.77 | -0.662 | (A)11.5, (R)9.1, (D) 8.1 |
| **ZmBBX4** | 27.72 | 5.19 | 37 | 26 | 3769 | 66.51 | 57.21 | -0.526 | (S)12.0, (A)10.9, (E)7.4 |
| **ZmBBX5** | 34.96 | 5.43 | 48 | 37 | 4673 | 41.61 | 63.12 | -0.382 | (A)19.4,(R)7.8, (G)7.5 |
| **ZmBBX6** | 28.09 | 7.55 | 31 | 32 | 3884 | 51.22 | 51.22 | -0.354 | (R)9.1, (G)8.7, (A)7.5 |
| **ZmBBX7** | 43.36 | 4.9 | 69 | 44 | 5978 | 52.08 | 68.9 | -0.417 | (A)16.9, (E)9.3, (G) 7.8 |
| **ZmBBX8** | 37.07 | 5.48 | 41 | 29 | 5181 | 56.55 | 66.27 | -0.248 | (A)17.0, (P)9.7, (G)7.8 |
| **ZmBBX9** | 28.35 | 5.73 | 30 | 26 | 3907 | 43.93 | 66.06 | -0.146 | (A)21.5, (S)11.1, (G)9.3 |
| **ZmBBX10** | 27.42 | 4.94 | 40 | 24 | 3742 | 44.07 | 73.48 | -0.318 | (A)15.8, (D)9.1, (L)7.9 |
| **ZmBBX11** | 33.04 | 5.41 | 47 | 35 | 4553 | 54.06 | 63.09 | -0.382 | (A)19.7, (S)8.9, (R)8.3 |
| **ZmBBX12** | 29.77 | 5 | 37 | 24 | 4006 | 64.01 | 67.87 | -0.226 | (A)16.2, (S)9.4, (P)7.9 |
| **ZmBBX13** | 51.43 | 7.53 | 55 | 56 | 7140 | 55.82 | 74.26 | -0.311 | (A)13.6, (R)8.5, (S)7.7 |
| **ZmBBX14** | 44.85 | 5.3 | 59 | 47 | 6113 | 54.23 | 67.09 | -0.52 | (S)11.1, (A)7.9 (L) 7.6 |
| **ZmBBX15** | 45.20 | 6.68 | 57 | 55 | 6295 | 59.74 | 64.58 | -0.623 | (A)10.4, (P)10.2, (R)9.0 |
| **ZmBBX16** | 37.46 | 5.19 | 46 | 35 | 5099 | 52.1 | 72.39 | -0.235 | (A)14.1, (D)9.6, (S)9.3 |
| **ZmBBX17** | 23.18 | 10.52 | 20 | 33 | 3208 | 62.06 | 67.83 | -0.208 | (A)26.5, (R)13.3, (S)7.5 |
| **ZmBBX18** | 35.52 | 5.04 | 51 | 34 | 4858 | 53.72 | 69.04 | -0.241 | (A)20.3, (E)9.0, (R)7.8 |
| **ZmBBX19** | 15.78 | 9.41 | 13 | 23 | 2148 | 84.88 | 70.99 | -0.343 | (A)15.5, (R)12.7, (P)8.5 |
| **ZmBBX20** | 51.16 | 5.33 | 66 | 52 | 7029 | 48.73 | 67.94 | -0.484 | (S)10.8, (D)8.5, (L) 8.2 |
| **ZmBBX21** | 50.71 | 5.84 | 68 | 62 | 6997 | 62.78 | 64.95 | -0.576 | (A)12.5, (R)8.8, (G)8.2 |
| **ZmBBX22** | 41.26 | 5.15 | 42 | 28 | 5725 | 62.51 | 72.49 | -0.311 | (S)12.2, (V)8.8 (A)8.5, |
| **ZmBBX23** | 28.58 | 4.55 | 40 | 25 | 3878 | 55.54 | 77.7 | -0.103 | (A)14.5, (D)12.3, (P)9.3 |
| **ZmBBX24** | 20.75 | 10.36 | 17 | 30 | 2899 | 63.94 | 72.24 | -0.193 | (A)24.4, (R)13.4, (G)9.5 |
| **ZmBBX25** | 48.91 | 5.24 | 68 | 53 | 6675 | 47.72 | 68.61 | -0.476 | (A)13.9, (G)9.5, (D)8.2 |
| **ZmBBX26** | 33.51 | 5.08 | 40 | 29 | 4621 | 50.18 | 60.5 | -0.355 | (A)14.3, (G)10.6, (S)9.0 |
| **ZmBBX27** | 22.35 | 5.42 | 32 | 23 | 3091 | 46.09 | 60.53 | -0.745 | (D)10.2, (P)6.8, (N) 6.3 |
| **ZmBBX28** | 45.94 | 5.27 | 45 | 33 | 6281 | 52.9 | 74.43 | -0.262 | (A)9.7,(S)9.5, (L)7.8 |
| **ZmBBX29** | 49.70 | 5.09 | 72 | 54 | 6793 | 49.28 | 64.74 | -0.561 | (A)12.1, (R)8.6, (L)8.1 |
| **ZmBBX30** | 46.84 | 5.56 | 49 | 38 | 6355 | 47.32 | 72.59 | -0.247 | (A)13.3, (S)10.0, (V)7.2 |
| **ZmBBX31** | 47.06 | 5.69 | 52 | 45 | 6501 | 49.79 | 69.2 | -0.337 | (S)12.6, (A)8.9, (D)7.6 |
| **ZmBBX32** | 38.44 | 5.81 | 49 | 38 | 5333 | 40.08 | 67.45 | -0.275 | (A)16.5, (G)8.2, (V) 8.0 |
| **ZmBBX33** | 28.21 | 6.08 | 27 | 25 | 3874 | 41.08 | 66.98 | -0.024 | (A)21.6, (S)10.8, (G) 29 |
| **ZmBBX34** | 26.51 | 4.54 | 44 | 23 | 3547 | 53.55 | 44.81 | -0.683 | (G)15.0, (A)12.7, (S)10.8 |
| **ZmBBX35** | 34.9 | 5.2 | 50 | 37 | 4672 | 38.37 | 65.88 | -0.329 | (A)20.4, (D)8.0, (R)7.4 |
| **ZmBBX36** | 27.67 | 4.8 | 41 | 25 | 3763 | 66.12 | 58.05 | -0.546 | (A)11.7, (S)10.5, (D)8.2 |
| **BdBBX1** | 46.55 | 5.69 | 63 | 53 | 6364 | 49.76 | 62.67 | -0.652 | (A)12.5, (D)9.9, (L) 8.3 |
| **BdBBX2** | 36.83 | 5.66 | 45 | 37 | 5090 | 52.08 | 71.09 | -0.149 | (A)17.0, (G)8.6, (R) 7.5 |
| **BdBBX3** | 43.58 | 5.03 | 58 | 41 | 4244 | 47.76 | 62.82 | -0.562 | (S)12.7, (D)9.0, (A)8.5 |
| **BdBBX4** | 41.7 | 5.71 | 47 | 38 | 5985 | 54.24 | 67.41 | -0.454 | (S)11.4,(A)10.8, (E)7.7 |
| **BdBBX5** | 47.9 | 5.72 | 68 | 61 | 5752 | 57.82 | 66.97 | -0.498 | (A)11.2, (R)9.6, (E)8.7 |
| **BdBBX6** | 39.0 | 5.12 | 43 | 28 | 6608 | 52.99 | 68.88 | -0.372 | (S)10.6, (A)9.8, (P)8.2 |
| **BdBBX7** | 41.04 | 5.87 | 48 | 40 | 5426 | 53.86 | 59.47 | -0.635 | (A)11.1, (S)7.9, (E)7.7 |
| **BdBBX8** | 37.6 | 5.4 | 42 | 32 | 5671 | 67.76 | 57.5 | -0.46 | (A)11.9, (P)11.1, (S)10.8 |
| **BdBBX9** | 35.03 | 4.97 | 42 | 29 | 5129 | 62.3 | 54.44 | -0.411 | (A)15.0, (P)10.2, (S)9.9 |
| **BdBBX10** | 39.82 | 5.73 | 49 | 41 | 4787 | 41.95 | 61.45 | -0.277 | (A)15.8, (G)11.6, (S)9.2 |
| **BdBBX11** | 23.7 | 5.08 | 35 | 25 | 5387 | 54.55 | 39.91 | -0.768 | (A)13.0,(G)13.9, (S)12.1 |
| **BdBBX12** | 52.67 | 5.62 | 64 | 55 | 3193 | 54.2 | 57.1 | -0.723 | (A)10.8, (S)9.2, (G)8.6 |
| **BdBBX13** | 27.32 | 4.93 | 39 | 24 | 7245 | 41.45 | 71.63 | -0.312 | (A)14.3, (D)8.9, (P) 7.8 |
| **BdBBX14** | 46.16 | 8.14 | 45 | 46 | 3838 | 47.06 | 72.54 | -0.399 | (A)9.9, (S)9.7, (R)8.0 |
| **BdBBX15** | 43.09 | 5.24 | 56 | 41 | 6393 | 52.41 | 61.4 | -0.555 | (S)12.0, (A)8.4, (L)7.4 |
| **BdBBX16** | 48.98 | 8.05 | 62 | 64 | 5891 | 66.63 | 63.53 | -0.579 | A)14.1, (G)10.7, (R)9.8 |
| **BdBBX17** | 23.9 | 6.03 | 28 | 22 | 6672 | 48.37 | 62.84 | -0.638 | (D)9.0, (S)8.1, (N) 7.6 |
| **BdBBX18** | 22.02 | 5.28 | 26 | 18 | 3151 | 50.13 | 67.43 | -0.278 | (A)15.3, (G)11.3, (R)7.2 |
| **BdBBX19** | 28.79 | 5.54 | 34 | 25 | 3101 | 56.3 | 70.34 | -0.24 | (A)15.4, (P)9.7, (S) 7.9 |
| **BdBBX20** | 35.85 | 5.64 | 49 | 38 | 3906 | 38.99 | 68.94 | -0.343 | (A)19.6, (G)9.7, (R) 7.6 |
| **BdBBX21** | 28.64 | 5.6 | 65 | 29 | 4932 | 63.09 | 65.99 | -0.401 | (A)16.1, (S)10.9, (E)7.9 |
| **BdBBX22** | 30.45 | 5.2 | 41 | 30 | 4101 | 62.65 | 73.36 | -0.376 | (A)13.5, (D)8.3, (S) 7.6 |
| **SbBBX1** | 45.96 | 6.16 | 60 | 55 | 6381 | 53.43 | 65.14 | -0.57 | A (11.7), P (9.8), R (8.8) |
| **SbBBX2** | 44.24 | 5.05 | 61 | 44 | 6064 | 53.49 | 59.42 | -0.63 | A (11.4), G (9.4%), P (8.0) |
| **SbBBX3** | 23.99 | 5.64 | 32 | 24 | 3151 | 49.42 | 58.67 | -0.703 | D (10.0), V (7.1), A (6.6) |
| **SbBBX4** | 43.93 | 4.99 | 59 | 38 | 5923 | 54.74 | 69.88 | -0.298 | A (16.7), G (9.9) E ( 8.2) |
| **SbBBX5** | 36.55 | 5.41 | 40 | 29 | 5013 | 52.09 | 63.75 | -0.305 | A (17.6), P ( 10.2), G (8.2) |
| **SbBBX6** | 28.56 | 4.94 | 39 | 24 | 3883 | 42.76 | 75.93 | -0.243 | A (17.9), D (8.4), L ( 7.6) |
| **SbBBX7** | 35.21 | 5 | 52 | 34 | 4829 | 43.7 | 65.98 | -0.274 | A (22), D (8.3), R (7.4) |
| **SbBBX8** | 52.89 | 5.59 | 71 | 61 | 7281 | 60.65 | 66.29 | -0.518 | A ( 13.3), E (8.4), R (8.2) |
| **SbBBX9** | 33.5 | 8.24 | 31 | 33 | 4606 | 54.38 | 78.93 | -0.421 | S (9.0), L (7.3), V (6.9) |
| **SbBBX10** | 30.77 | 5.38 | 30 | 23 | 4151 | 60.5 | 61.76 | -0.254 | A (15.6), S ( 13.9), G ( 9.5) |
| **SbBBX11** | 28.9 | 4.93 | 36 | 24 | 3922 | 48.09 | 72.24 | -0.198 | A (14.6), P (9.3), S (7.8) |
| **SbBBX12** | 34.41 | 5.19 | 49 | 36 | 4737 | 41.62 | 64.68 | -0.32 | A (20.4), G (8.2), D (7.9) |
| **SbBBX13** | 28.11 | 4.91 | 40 | 25 | 3821 | 66.58 | 58.52 | -0.469 | A (12.1), S (12.1), G ( 8.0) |
| **SbBBX14** | 27.42 | 4.66 | 47 | 24 | 3673 | 52.35 | 42.32 | -0.75 | G (15.5), A (13.7), E (10.0) |
| **SbBBX15** | 51.87 | 6.51 | 61 | 58 | 7127 | 48.75 | 65.27 | -0.48 | A (13.3), G (9.6), S (8.8) |
| **SbBBX16** | 24.91 | 5.03 | 28 | 15 | 3331 | 60.55 | 58.24 | -0.293 | S (13.9), G (13.1), A (11.4) |
| **SbBBX17** | 34.68 | 5.14 | 39 | 28 | 4761 | 60.69 | 58.57 | -0.349 | A (14.9), G (10.7), P (10.1) |
| **SbBBX18** | 40.69 | 5.02 | 42 | 27 | 5521 | 57.84 | 67.89 | -0.338 | S (10.1), A (9.9), V (8.5) |
| **SbBBX19** | 50.89 | 5.2 | 71 | 54 | 6943 | 51.5 | 66.88 | -0.483 | A ( 14.3), G (9.9), L ( 8.2) |
| **SbBBX20** | 44.36 | 5.38 | 47 | 37 | 6080 | 50.76 | 71.31 | -0.3 | A (13), S (11), V(8) |
| **SbBBX21** | 43.61 | 5.22 | 53 | 43 | 5993 | 50.61 | 66.6 | -0.469 | S ( 13.5), A (8.6), D (8.4) |
| **SbBBX22** | 40.62 | 5.97 | 49 | 38 | 5495 | 41.78 | 68.14 | -0.312 | A (16.2), G (8.5), V(8.2) |
| **SbBBX23** | 31.42 | 4.63 | 46 | 28 | 4367 | 45.73 | 70.85 | -0.209 | A (16.1), D (12.1), S ( 8.2) |
| **SbBBX24** | 45.96 | 6.16 | 60 | 55 | 6381 | 53.43 | 65.14 | -0.57 | A (11.7), P (9.8), R (8.8) |
| **SiBBX1** | 39.46 | 6.32 | 41 | 38 | 5435 | 42.57 | 67.51 | -0.112 | A ( 21.2), G (10.9), V (8.3) |
| **SiBBX2** | 28.29 | 5.52 | 30 | 24 | 3860 | 56.54 | 63.72 | -0.251 | A (16.4), S (11.5), G (7.4) |
| **SiBBX3** | 44.61 | 4.99 | 61 | 44 | 6076 | 58.69 | 58.08 | -0.613 | S (11.8), D (9.8), A (7.9) |
| **SiBBX4** | 39.79 | 8 | 34 | 36 | 5487 | 45.47 | 68.42 | -0.445 | S (10.7), A (9.3), V (7.9) |
| **SiBBX5** | 50.22 | 5.75 | 69 | 61 | 6947 | 63.35 | 66.26 | -0.526 | A ( 14.2), R ( 8.8), E(8.0) |
| **SiBBX6** | 43.63 | 5.2 | 51 | 41 | 5966 | 48.13 | 63.72 | -0.476 | S ( 13.8), A (9.1), D (8.4) |
| **SiBBX7** | 19.35 | 5.88 | 23 | 17 | 2623 | 43.41 | 57.23 | -0.801 | D (9.0), S (9.0), G (8.5) |
| **SiBBX8** | 33.97 | 5.29 | 37 | 28 | 4658 | 52 | 61.87 | -0.272 | A (16.0), G (9.2), P (9.2) |
| **SiBBX9** | 22.29 | 5.55 | 24 | 17 | 3041 | 52.25 | 69.91 | -0.104 | A (12.8), G (11.9), S ( 10.6) |
| **SiBBX10** | 40.23 | 5.18 | 40 | 26 | 5514 | 57.77 | 66.38 | -0.33 | A ( 11.4), S (10.6), P(8.2) |
| **SiBBX11** | 47.59 | 5.09 | 67 | 51 | 6566 | 50.48 | 65.93 | -0.467 | A (14.4), G (9.4), R (8.3) |
| **SiBBX12** | 39.57 | 6.13 | 46 | 38 | 5438 | 46.68 | 67.34 | -0.259 | A (17.5), G (7.8),V ( 7.8) |
| **SiBBX13** | 31.09 | 5.12 | 38 | 27 | 4267 | 52.66 | 76.68 | -0.106 | A (15.9), S (10.3), D (9.3) |
| **SiBBX14** | 42.83 | 5.89 | 40 | 32 | 5924 | 57.33 | 67.82 | -0.202 | A (17.4), P (12.3), S (8.2) |
| **SiBBX15** | 27.21 | 4.79 | 37 | 23 | 3756 | 42.78 | 70.12 | -0.266 | A ( 14.0), P (9.3), D (8.1) |
| **SiBBX16** | 34.39 | 5.15 | 50 | 37 | 4730 | 41.32 | 67.36 | -0.296 | A (20.6), R (7.7), D (7.7) |
| **SiBBX17** | 27.89 | 4.98 | 39 | 26 | 3798 | 65.39 | 63.68 | -0.428 | A (13.2), S (10.1), E (8.1) |
| **SiBBX18** | 44.45 | 6.65 | 57 | 55 | 6171 | 55.8 | 66.18 | -0.588 | A (11.3), R (9.6), P (9.6) |
| **SiBBX19** | 69.18 | 8.49 | 69 | 76 | 9554 | 68.81 | 59.89 | -0.617 | A (11.7), P ( 10.6), S (9.7) |

**Table S1**. **Abbreviations:** MW, molecular weight; pI, isoelectric point; -VE CR, negative charge residues; +VE CR, positive charge residues; Ii, index instability; Ai, aliphatic index; GRAVY, grand average of hydropathicity; (A, Ala; P, Pro; S, Ser; G, Gly; L, Leu:N, ASN; T,Thr). The information about protein was taken from ExPASy (http://web.expasy.org/protparam/)

**Table S2:** The sequence analysis and weblog of 10 identified motifs of the BBX gene family in five *Poaceae* species.

| **S. No** | **Sequences** | **Width** | **Sites** | **Logo** |
| --- | --- | --- | --- | --- |
| 1 | RRFDKKIRYASRKAYAETRPRIKGRFAKR | 29 | 70 | 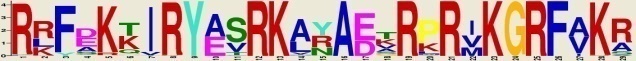 |
| 2 | AVYCCADEAALCAACDRDVHSANPLARRHERVPLLPPCAAA | 41 | 126 | 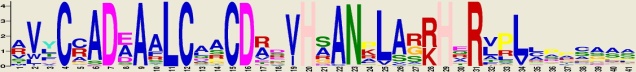 |
| 3 | PLCDICQEKRAYFFCVEDRAJLCRDCDVAVHTAN | 34 | 58 | 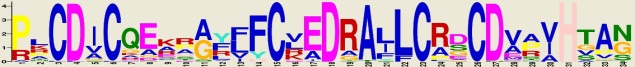 |
| 4 | EREARLSRYREKRKT | 15 | 68 | 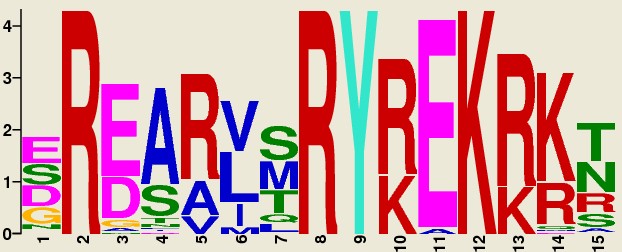 |
| 5 | ALVSRHRRFLLTGVRVGLAPA | 21 | 44 | 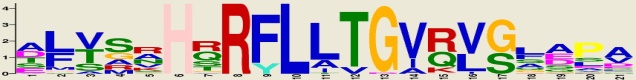 |
| 6 | VLCDVCEAAPA | 11 | 115 | 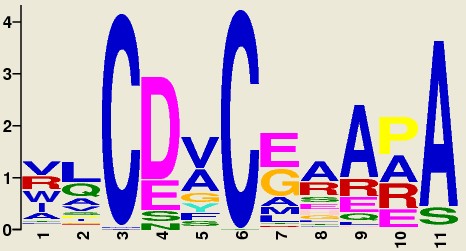 |
| 7 | GHKRQTINCYSGCPSSAELSRIWSFVMDIPTVAPEPNCEQGISMMSISDS | 50 | 10 | 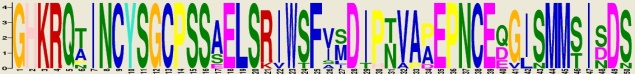 |
| 8 | IYEDFCVDDADLTFENYEELFGTSHIQTEZLFDDAGIDSYFEMK | 44 | 11 | 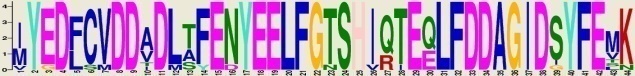 |
| 9 | SLSFSGLTGESSAGDHQDCGVSPMLLMGEPPWHPP | 35 | 11 | 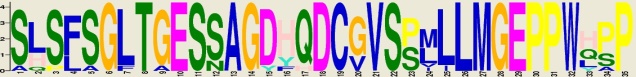 |
| 10 | LPGWHVEDFLVDSAY | 15 | 36 | 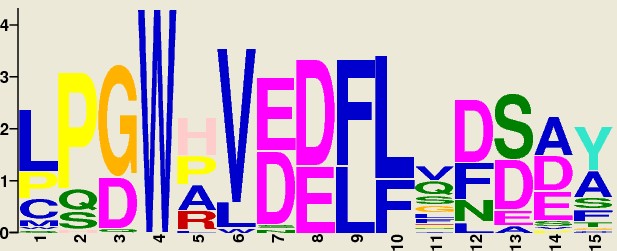 |
|  |  |  |  |  |

**Table S3:** List of the primers used during this study.

| **Name** | **Forward primer** | **Reversed primer** |
| --- | --- | --- |
| *OsBBX1* | CCTCCTCTCCTCCTCCTCTT | TCCTCGACGCAGAAGAAGTA |
| *OsBBX2* | CGCGTTTCTTTGCTTGGTAT | TCATCCTCCTCATCCACCAC |
| *OsBBX7* | TCGTAATGTGCATTCAGCCA | TCGTAATGTGCATTCAGCCA |
| *OsBBX8* | GCAGGACTCGTTCTACATGG | GCAGGACTCGTTCTACATGG |
| *OsBBX9* | CGGCGATCATCAGTTACCTT | CGGCGATCATCAGTTACCTT |
| *OsBBX12* | CTCCATGGACGACATCAAGG | CTCCATGGACGACATCAAGG |
| *OsBBX14* | ATTACCACCACCTCCTCCTC | ATTACCACCACCTCCTCCTC |
| *OsBBX16* | TCAAGTTGGCCTTGATCCTG | TCAAGTTGGCCTTGATCCTG |
| *OsBBX17* | GAGATGCTGGACATCGACTT | GAGATGCTGGACATCGACTT |
| *OsBBX19* | TGGTCATAAGAGGCAGACCA | TGGTCATAAGAGGCAGACCA |
| *OsBBX21* | CGGTGGCGTCGTCGATGG | TGGGCGCTTCAGAACACGAG |
| *OsBBX24* | GACGAGGACGAGGAGGTGGT | CACGGCTGGTCCTCCTTGAC |

**Figure S1**: Transmembrane activity of the BBX genes members in maize, rice, sorghum, brome and millet. (A) Maize, (B) rice, (C) Sorghum, (D) brome, (E), millet, respectively.

**(A)**

**
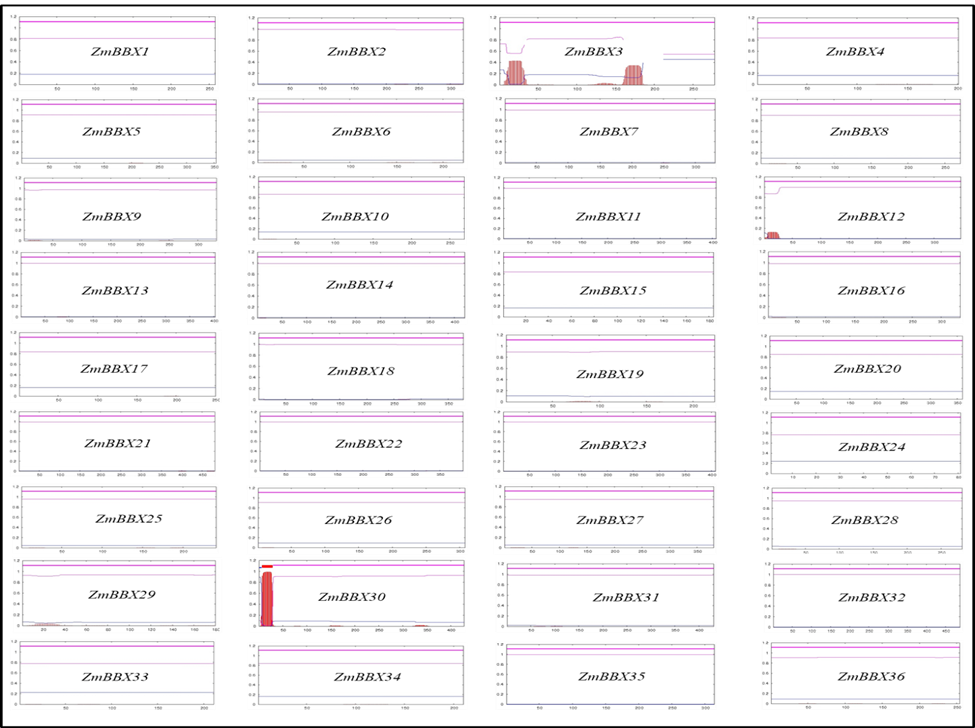
**

**(B)**


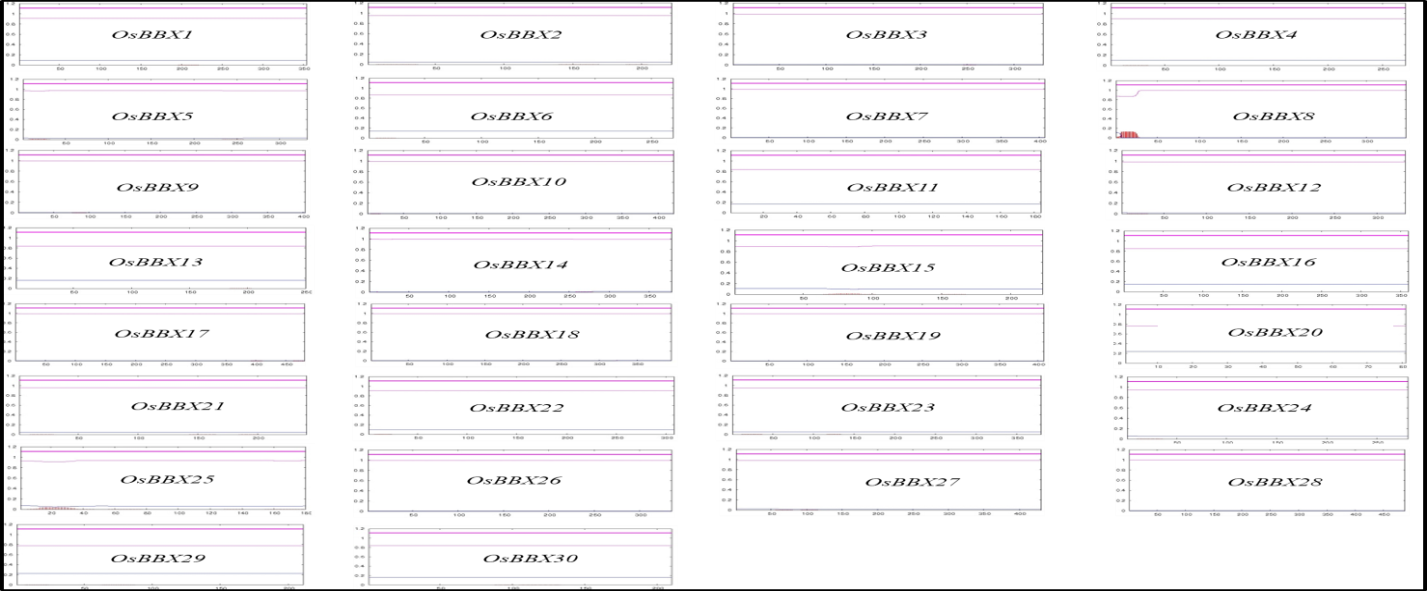


**(C)**

**
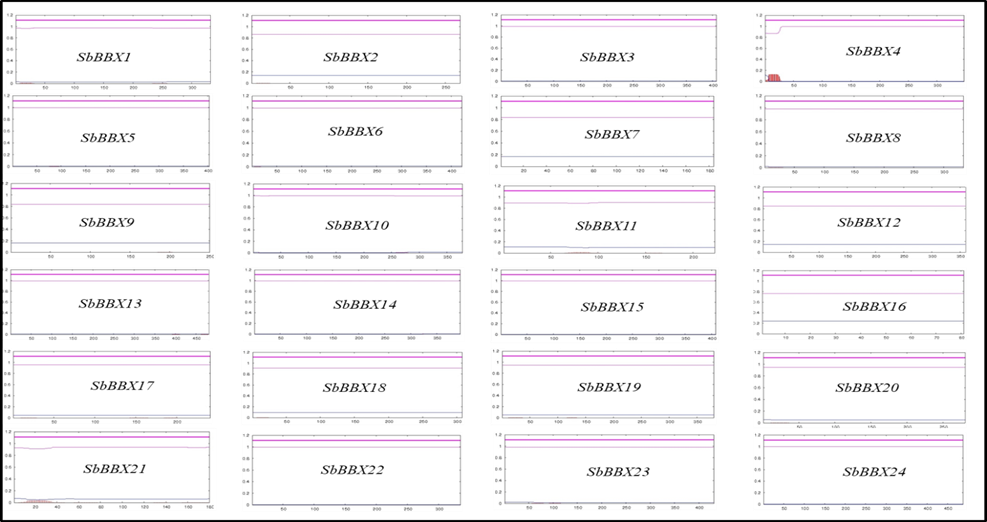
**

**(D)**


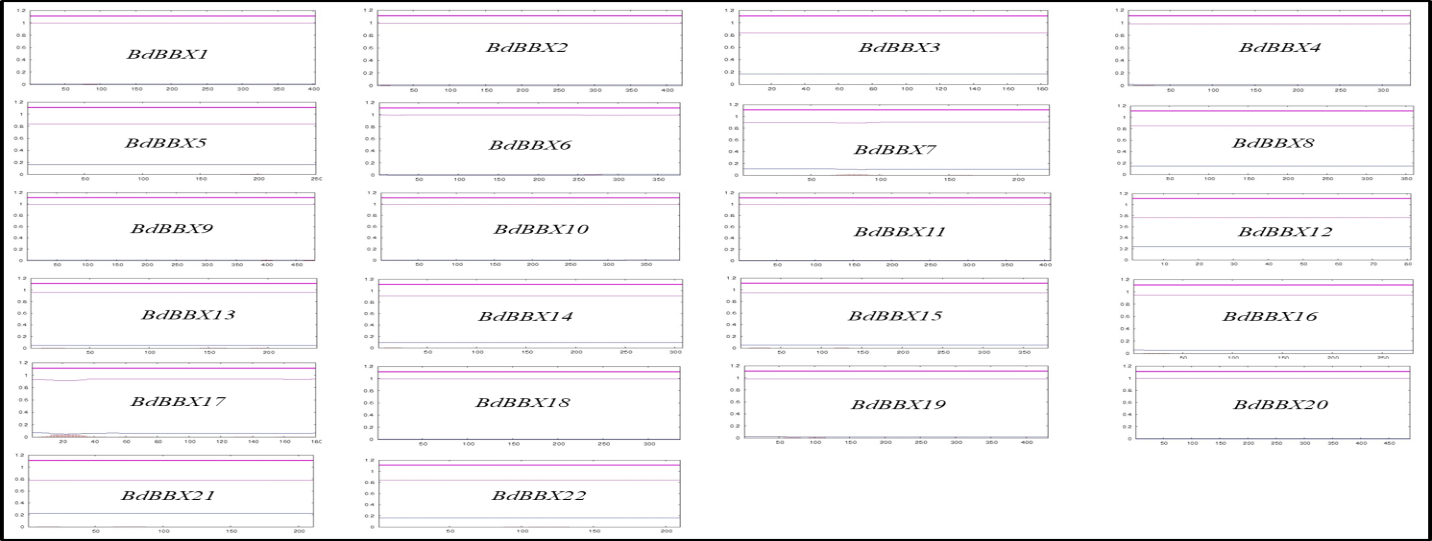


**(E)**

**
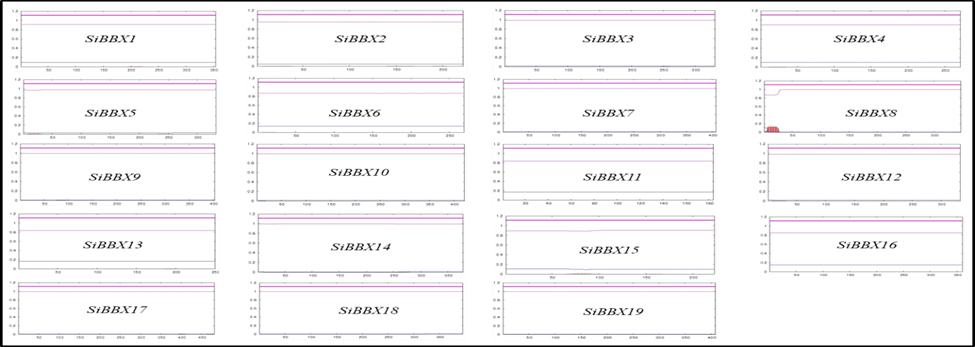
**

**Figure S2.** The exon/intron structures and identified motifs of BBX family genes. The structure of individual *BBX* gene was obtained through the Gene Structure Display Server (http://gsds. cbi.pku.edu.cn) by aligning the coding or cDNA sequences with their corresponding genomic DNA sequence. 10 motifs were identified through online meme tool. (A) Maize, (B) rice, (C) Sorghum, (D) brome, (E), millet, respectively.

**(A)**

**
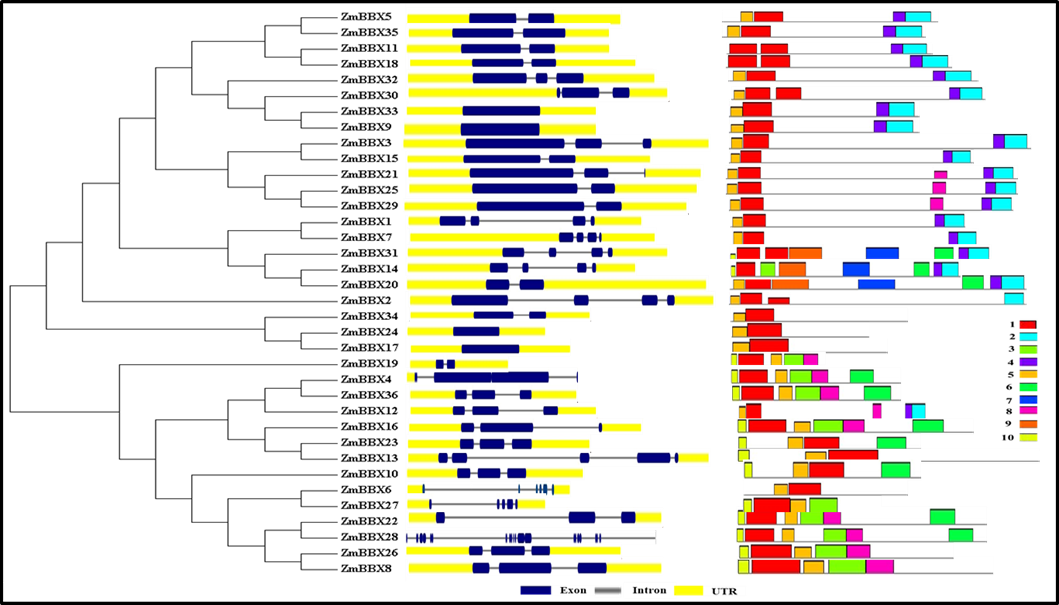
**

**(B)**


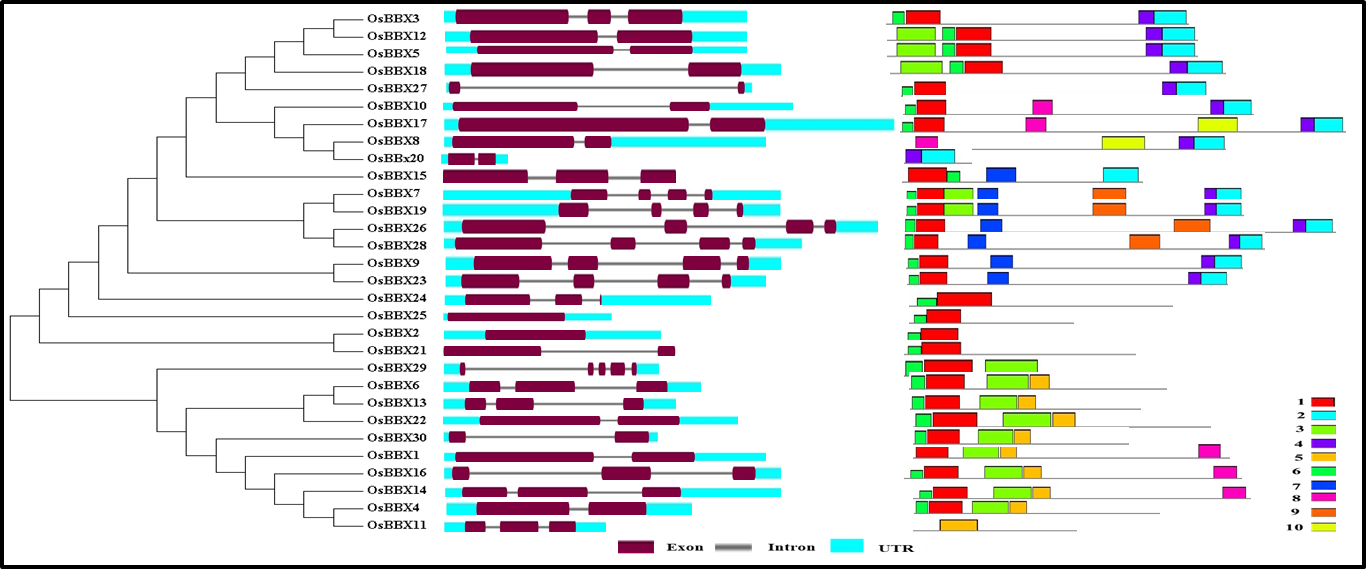


**(C)**


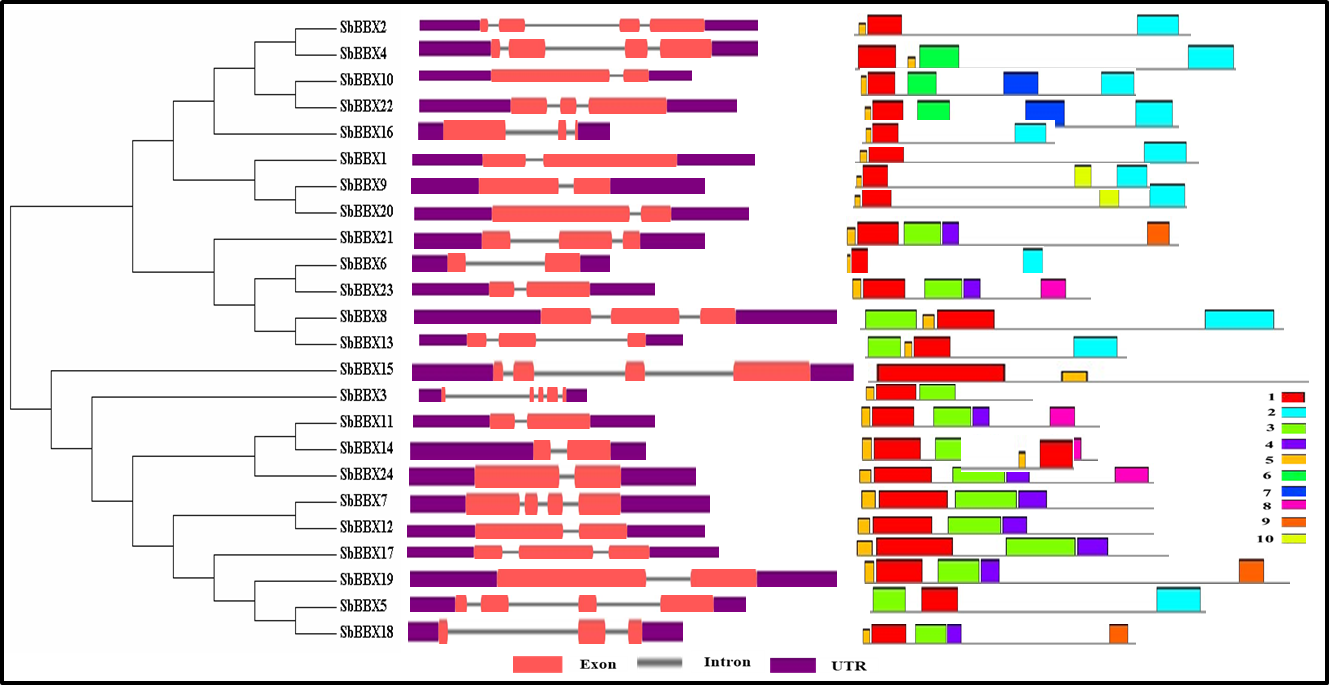


**(D)**


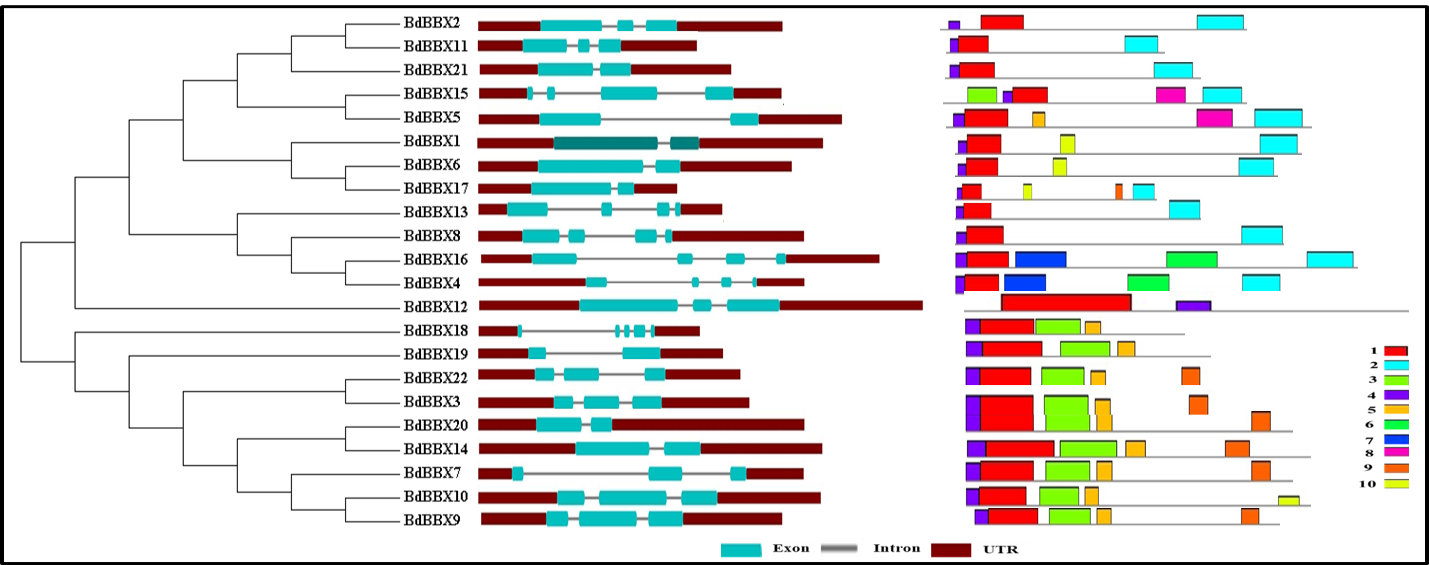


**(E)**


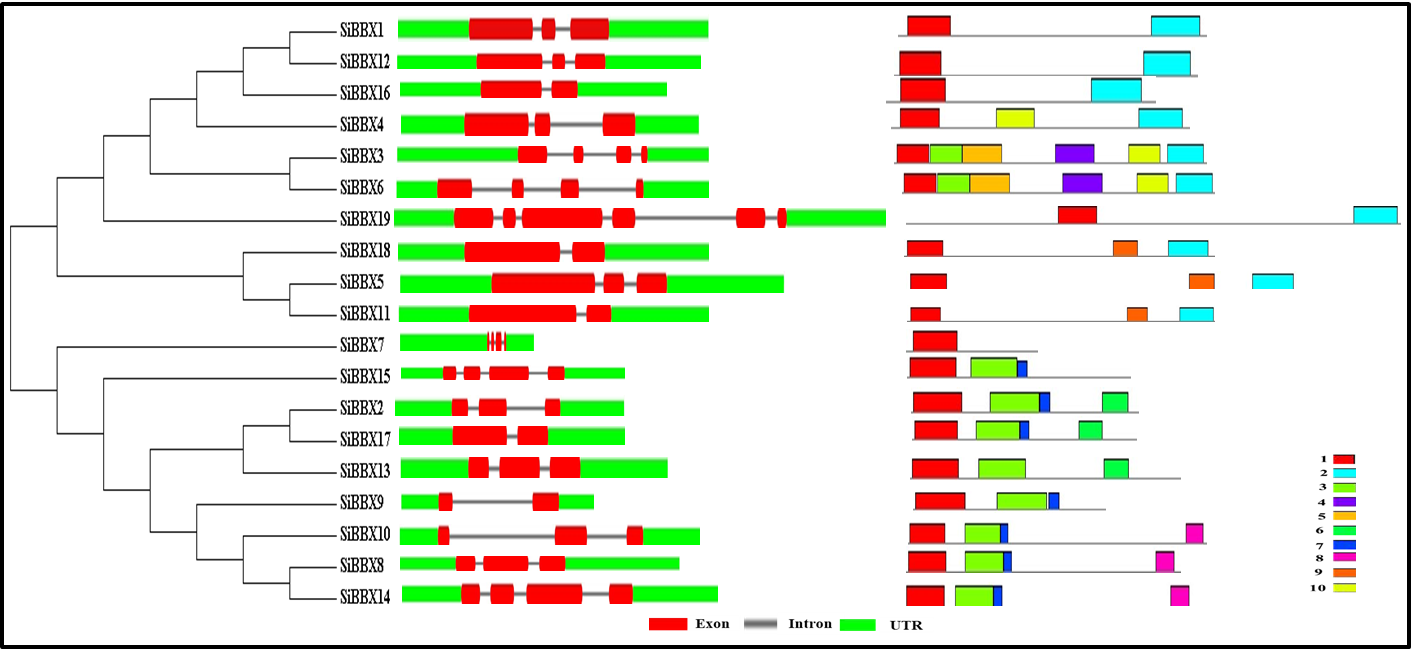


**(A)**


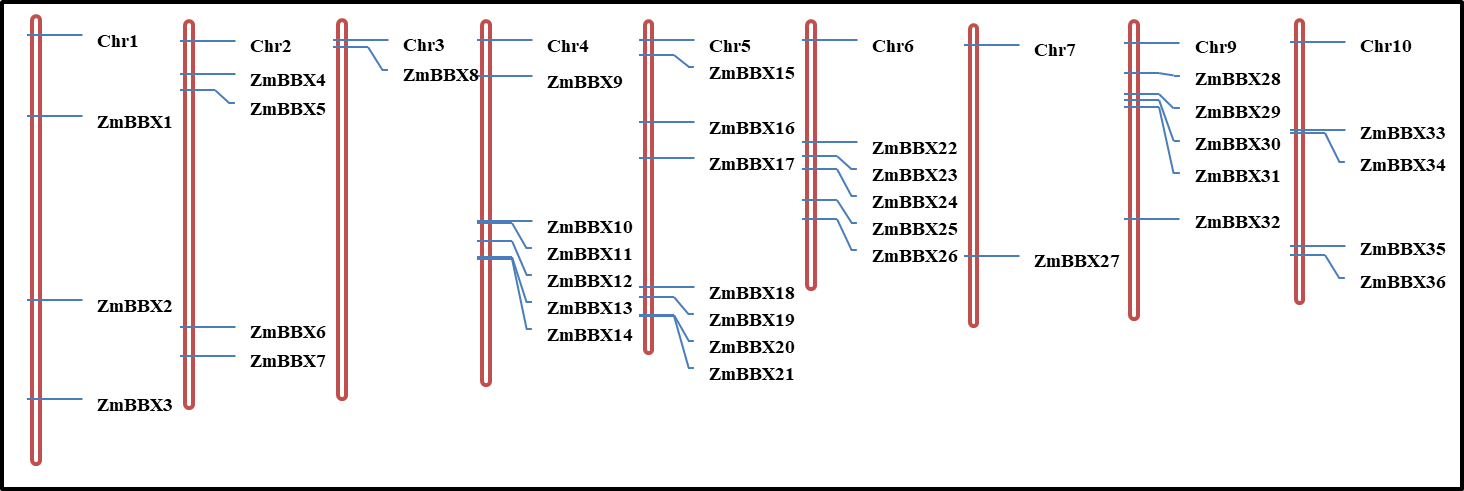


**(B)**

**
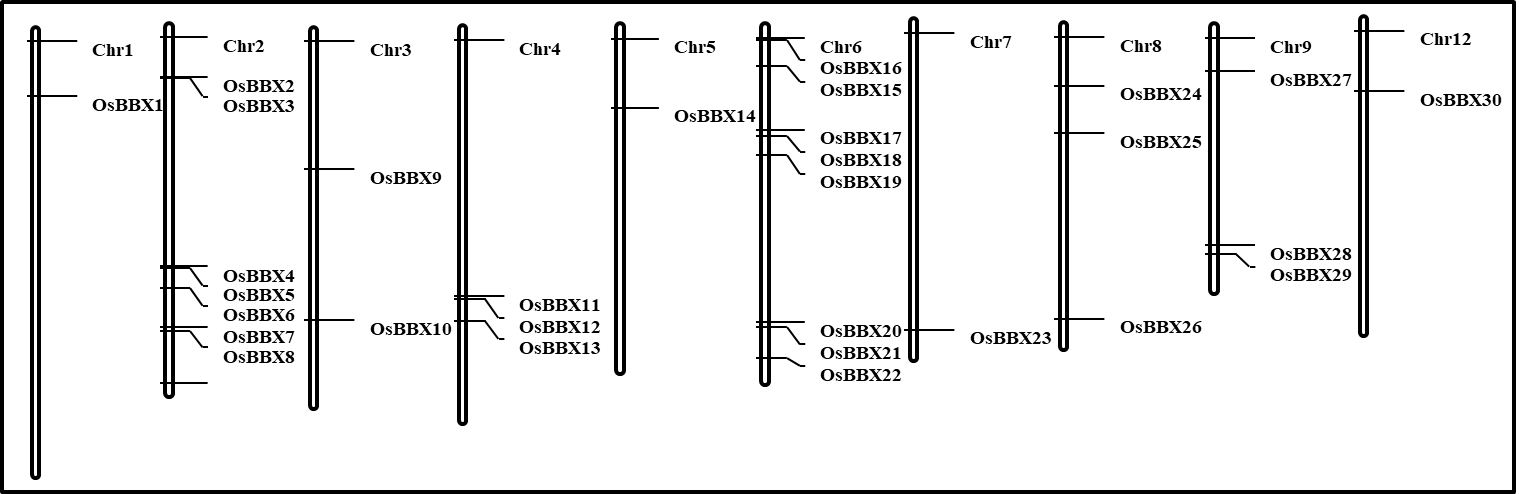
**

**(C)**


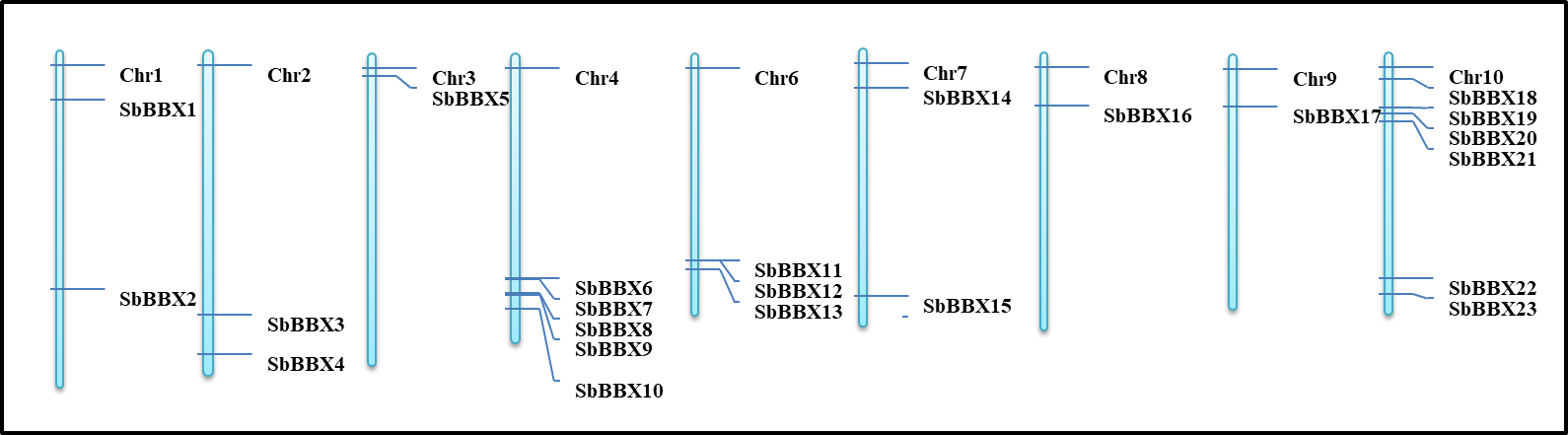


**(D)**


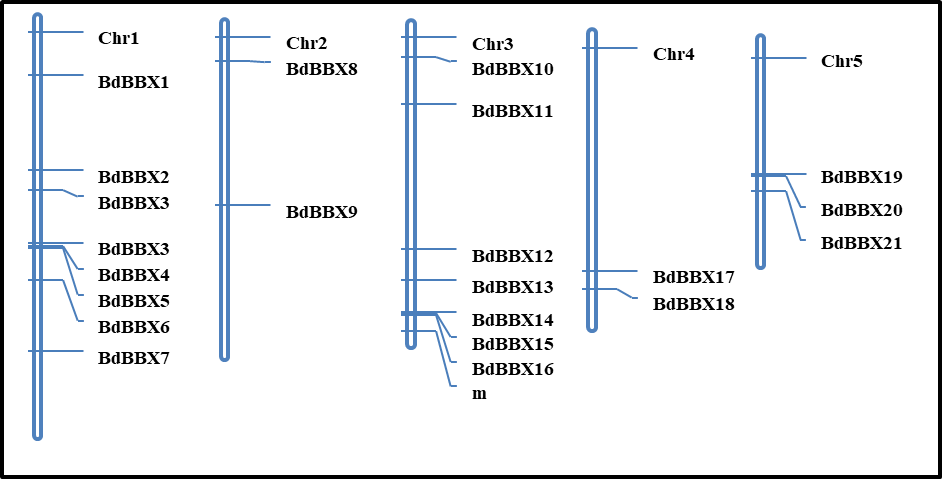


**(E)**


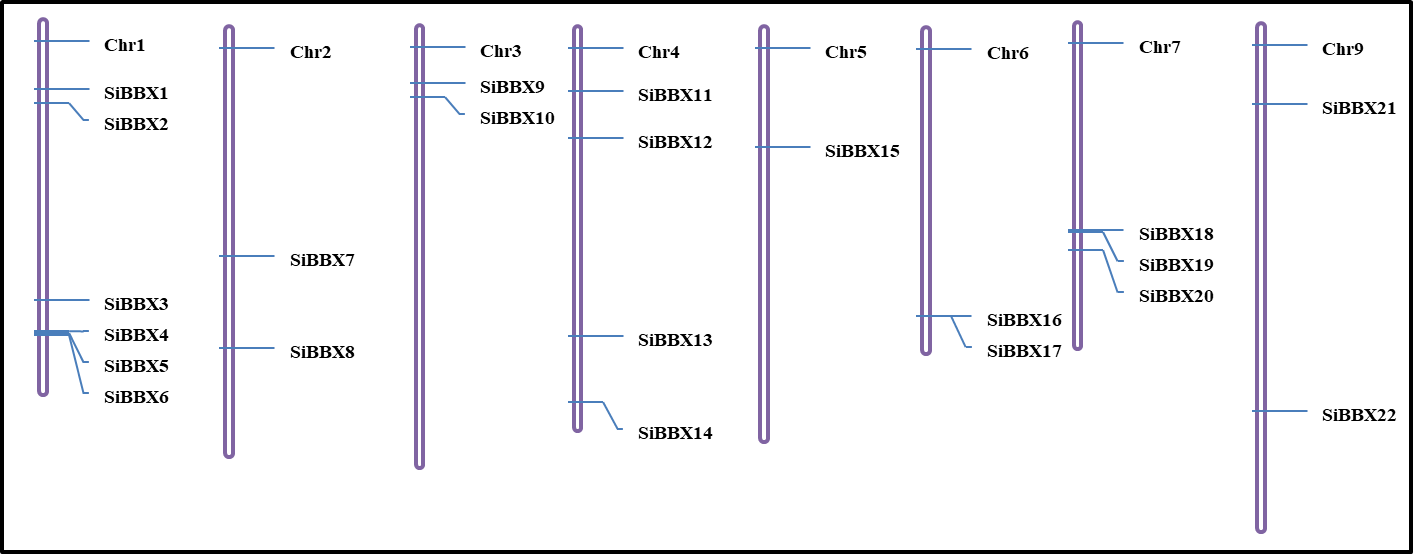


**Figure S3:** Chromosomal location of BBX genes on *Poaceae* chromosomes. (A), maize; (B), rice; (C), sorghum*;* (D), stiff brome, (E), millet, respectively. The graphical view was drawn from each gene ID and scaffolds information and position of each gene are indicated by line.


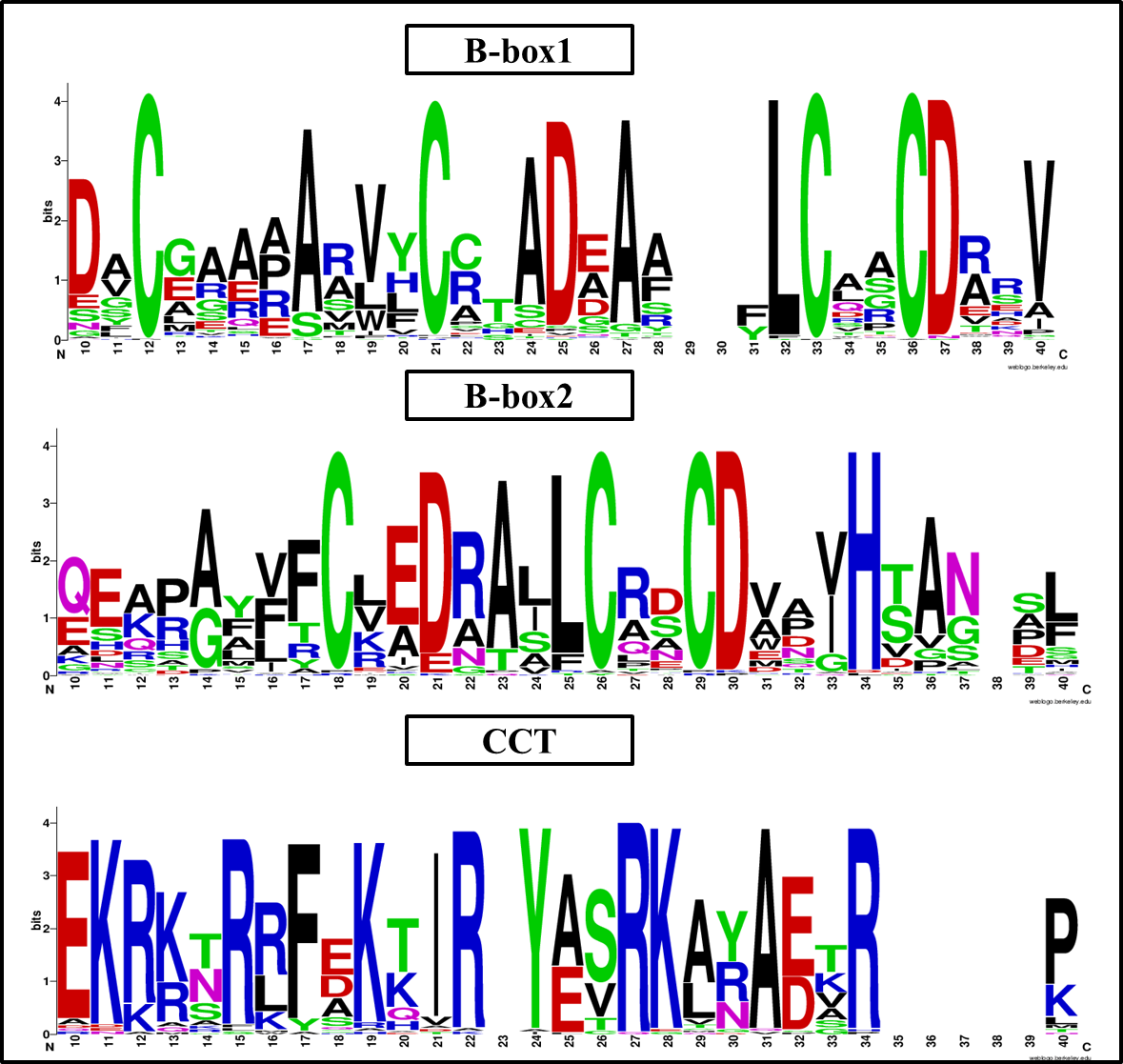


**Figure S4**: The web-logos B-Box 1, B-Box2 and CCT-domain of *Poaceae* BBX members.

**Figure S5a:** Detail alignment sequences of B-BOX1 domain of BBX gene family of five *Poaceae* species.


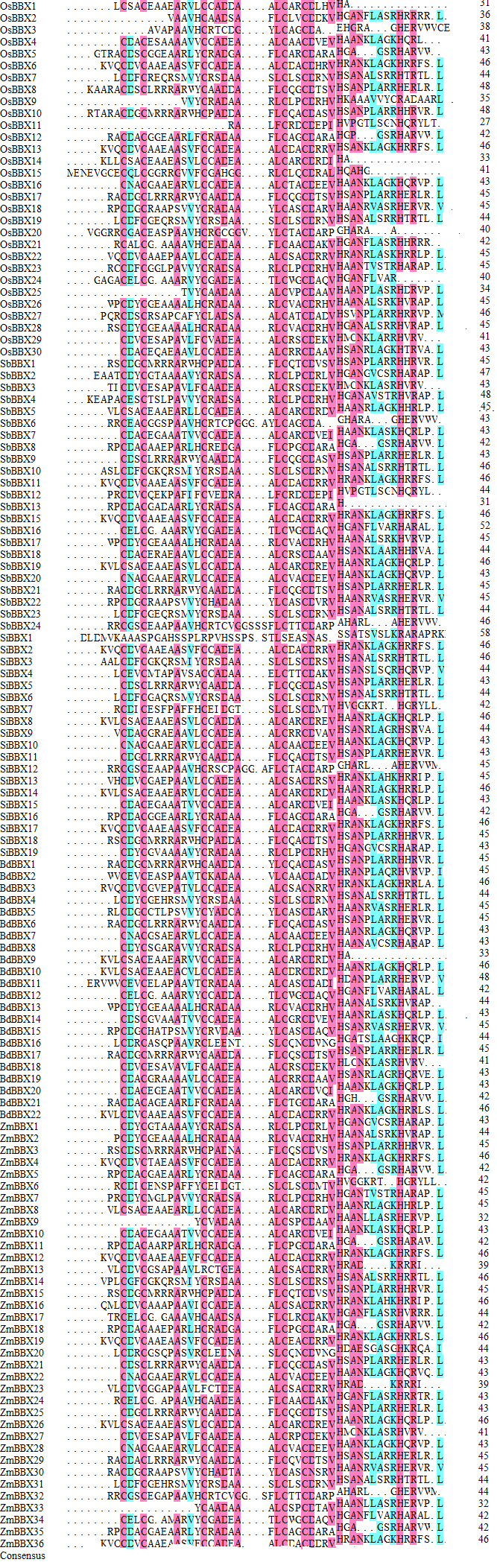


**Figure S5b:** Detail alignment sequences of B-BOX2 domain of BBX gene family of five *Poaceae* species.


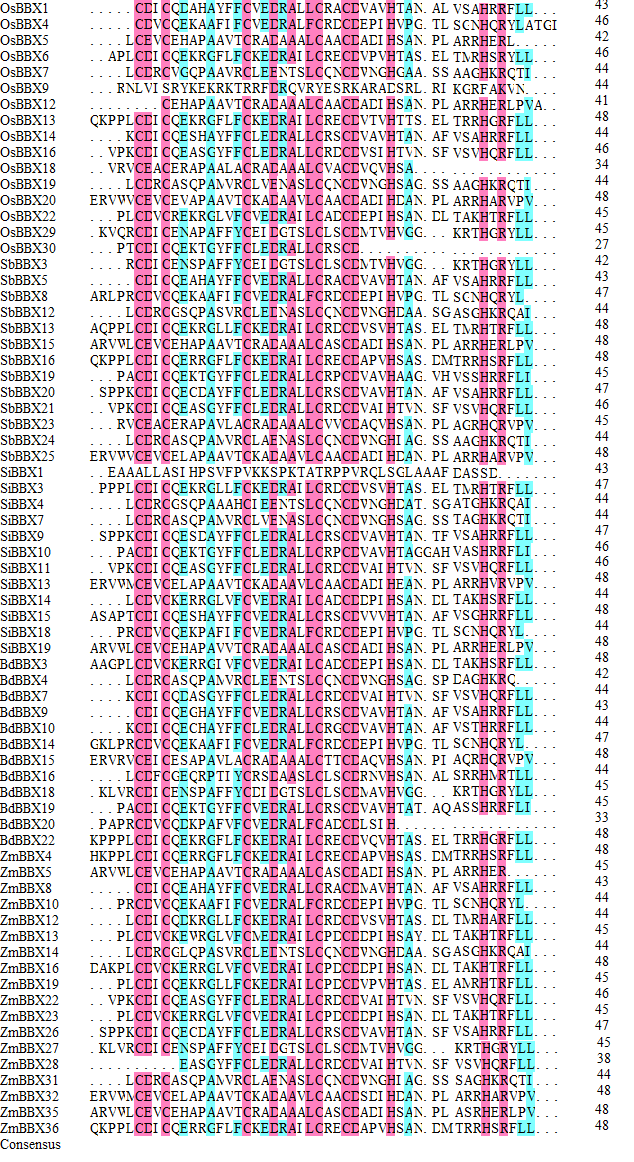


**Figure S5c:** Detail alignment sequences of CCT domain of BBX gene family of five *Poaceae* species.


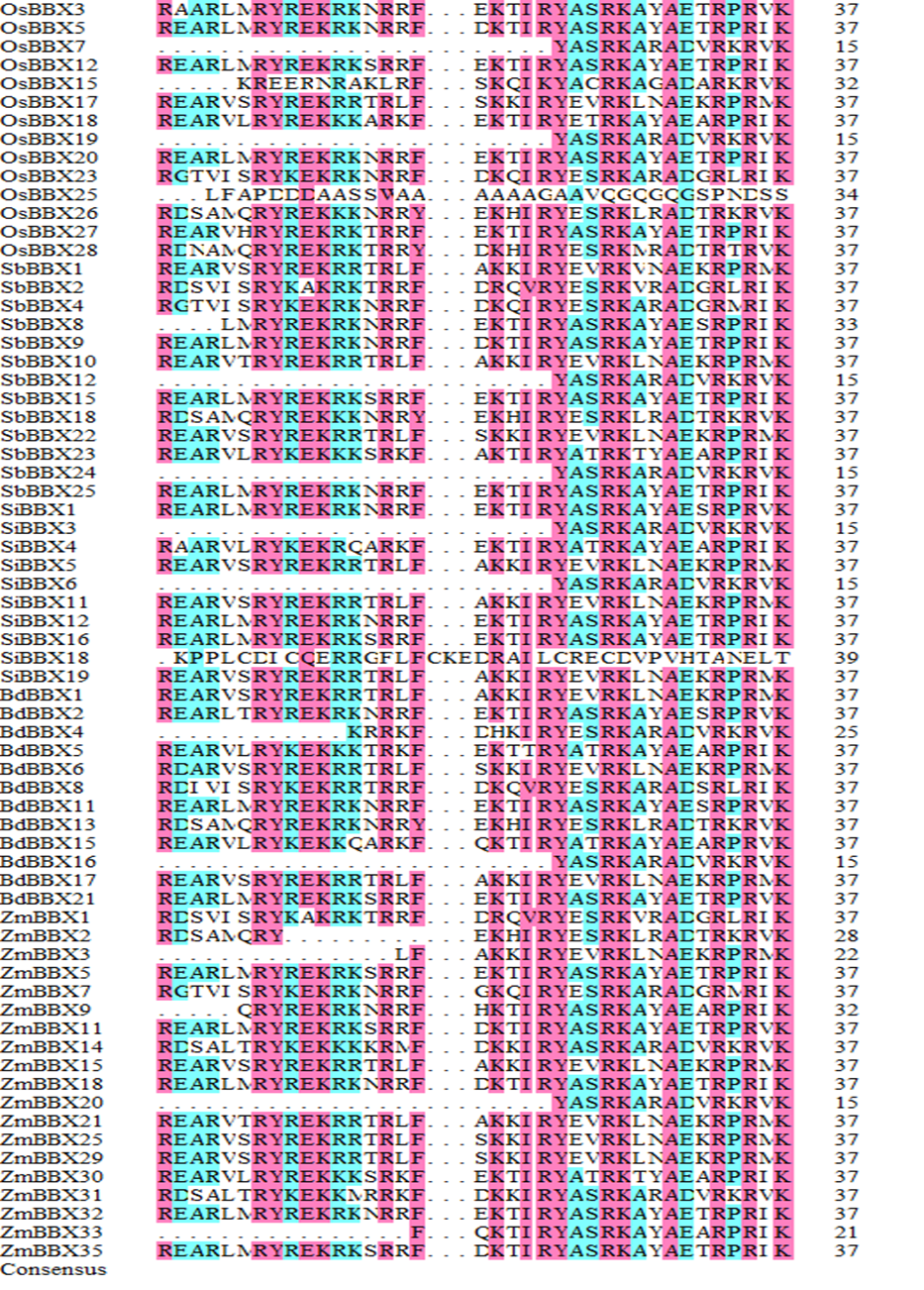

Supplement: Supplementary file 1 — Supplementary Figures and Supplementary tables. (DOCX 5100 kb) [file 12864_2018_5336_MOESM1_ESM.docx]
